# Elucidating Sodium-Ion Storage Mechanisms in Bowl-Shaped Hierarchical Porous Carbon Anodes: A Combined Solid-State NMR and EPR Approach

**DOI:** 10.1021/jacs.6c08138

**Published:** 2026-09-08

**Authors:** Ananya Pal, Nicole Leifer, Daphna Shimon

**Affiliations:** † Institute of Chemistry, The Hebrew University of Jerusalem, Jerusalem 9190401, Israel; ‡ Department of Chemistry, Institute of Nanotechnology and Advanced Materials, Bar-Ilan University, INIES—Israel National Institute for Energy Storage, Supported by Israel Ministry of Energy and Infrastructures, Ramat Gan 5290002, Israel

## Abstract

Understanding sodium-ion storage in carbon anodes is essential for fabricating efficient batteries via the design of carbon architectures with optimized pores, defects, and surface area. Previous mechanistic studies have mainly focused on hard carbon anodes with poorly defined structures, leaving the structural correlation with sodium storage unexplored. This work presents an investigation of the Na^+^ ion storage mechanisms in bowl-shaped hierarchical porous carbon anodes using a combination of nuclear magnetic and electron paramagnetic resonance spectroscopies. The material exhibits hierarchical porosity, a surface area of 917.5 m^2^/g, and delivers a reversible capacity of 250 mAh/g at 0.1 A/g in 1 M aqueous NaClO_4_. Solid-state magic-angle spinning nuclear magnetic resonance is used to identify four distinct Na^+^ ion environments in the carbon anode material. In addition, temperature-dependent continuous-wave electron paramagnetic resonance spectroscopy enables the differentiation between weakly adsorbed Na^+^ ions and more strongly interacting Na^+^ ions by analyzing line widths and g values as the cell voltage is varied. Our results reveal the correlation of the sodium storage processes: adsorption, insertion, and intercalation, to the potential of the electrochemical cell. This work establishes a clear relationship between hierarchical carbon architecture and Na^+^ storage pathways as a function of potentials. Providing mechanistic insight will help design advanced carbonaceous anodes for efficient sodium batteries.

## Graphical abstract

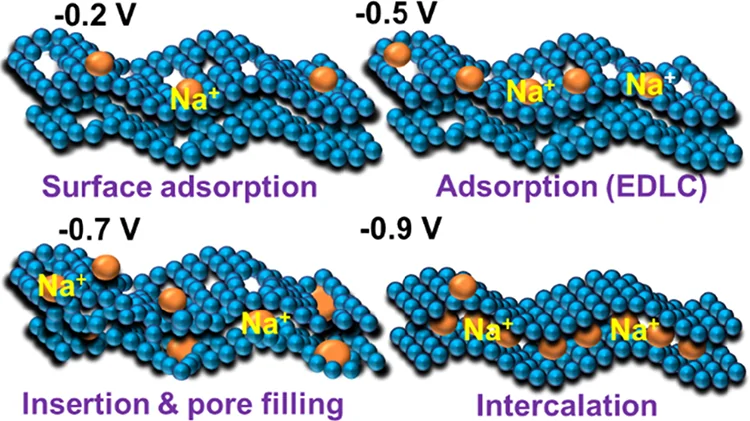

## 1 Introduction

The pressing need for sustainable, effective energy storage solutions to meet the escalating demand for clean energy has driven extensive research into battery technologies. Aqueous sodium-ion batteries (ASIBs) have garnered considerable attention owing in emphasize safety, environmental benignity, and cost-effectiveness.^1,2^ Water, as an electrolyte, possesses unique physicochemical properties, including intrinsic safety, high ionic conductivity, strong ion-solvation capability, and broad compatibility with alkali and alkaline-earth metal ions, endowing aqueous batteries with distinctive electrochemical characteristics.^3^ Moreover, the natural abundance of sodium, its low cost, and the compatibility of sodium-based systems with existing industrial infrastructure make ASIBs scalable energy storage systems.^4^ Though ASIBs possess highly promising features, their practical application remains limited due to several challenges. One of the major challenges is the development of suitable anode materials, as it has been found that graphite, the state-of-the-art anode material for lithium-ion batteries, is not suitable for sodium-ion storage due to the larger ionic radius of Na^+^ and its limited ability to intercalate into graphite layers without cointercalants.^5^ By contrast, nongraphitizable hierarchical porous carbons have played a significant role as anode materials for SIBs due to their exclusively tunable mechanical and electronic properties, ample interlayer space, high surface area, and diverse porosity.^6^ These porous carbons possess numerous surface defects, functional groups, and turbostratically aligned graphene layers, which collectively generate a hierarchical network of micro-, meso-, and macropores.^7^ In addition, the presence of structural defects and the high degree of disorder facilitate rapid sodium-ion transport, thereby improving battery performance in ASIBs.

Understanding sodium storage mechanisms in carbonaceous anode materials remains an active field of research due to the complexity of the carbon framework, particularly when hierarchical porosity is present. There are currently three proposed models of Na^+^-carbon interaction: Dahn et al. introduced the “insertion-absorption” model: Na^+^ ions initially intercalate into graphitic layers during the sloping region of the voltage profile, and then adsorb onto defect and pore sites, as indicated by the plateau region.^8,9^ In contrast, Cao et al. proposed the **“**adsorption-insertion” model, in which the sloping region is associated with Na^+^ adsorption on surface sites, whereas the plateau arises from Na^+^ intercalation into graphitic-like domains.^10^ Bommier et al. further showed that after the intercalation process, Na^+^ ions occupy the pores by adsorbing onto their surfaces, particularly toward the end of the plateau region.^11^ Thus, it is accepted that the Na^+^ ion storage mechanism within carbon frameworks proceeds through distinct stages: adsorption at defect sites and edges of the carbon framework, intercalation into the interlayer spaces between graphene layers, and insertion into the nanopores of the structure. However, the exact mechanism of sodium storage in ASIBs remains an open research question.

A robust understanding of the distribution of the Na^+^ ions at the electrode-electrolyte interface is quite challenging, especially in disordered, hierarchically structured carbons. However, this phenomenon is a critical element of the energy storage performance of the carbonaceous material.^12^ Solid-state magic-angle spinning nuclear magnetic resonance (MAS NMR) has been successfully employed to investigate the behavior of molecules/ions in porous carbons. The chemical environment of the ions/molecules within the hierarchical porous carbon framework (i.e., graphene layers, inside interstitial sites, graphene voids, and nanopores) is directly correlated with the observed chemical shift in MAS NMR and enables them to be distinguished from those residing in mesopores, macropores, or on the external surface.^13,14^ Lee et al. used low-temperature solid-state NMR to probe ion sorption within layered graphene galleries, thereby distinguishing ions at in-pore versus ex-pore sites.^13^ Further, Lyu et al. studied the sorption of aqueous electrolytes in porous carbon using NMR spectroscopy, providing experimental evidence of H_3_O^+^ uptake in the pores.^15^ The ^23^Na chemical shift is highly sensitive to the degree of ion confinement and the local electronic structure of the carbon framework, enabling discrimination between Na^+^ ions that are weakly adsorbed on pore surfaces, confined within nanopores, or intercalated between graphene layers.

The hierarchical porous carbon framework contains many topological defects, dangling bonds, and turbostratically stacked graphene layers, which give rise to intrinsic paramagnetism.^16^ This same heterogeneous pore structure also facilitates surface-dominated charge storage.^17^ In order to gain a better understanding of the electronic/structural properties of the carbon material, and its impact on electrochemical performance, electron paramagnetic resonance (EPR) spectroscopy can be an excellent complementry tool to MAS NMR. EPR is inherently much more sensitive. This makes it well-suited for detecting even trace concentrations of paramagnetic species, and providing insight into the electronic environment, including the identification of defect states and spin–spin interactions in these kinds of materials.^18^ Variations in signal intensity, line width, and *g*-value reflect changes in spin density, degree of graphitization, and local bonding environments. For example, Wang et al. explored how EPR line shapes and *g*-values can distinguish between different structural and electronic states in hard carbon anodes for Na-ion batteries.^19^

In this work, we synthesized conjugated, bowl-shaped hierarchical porous carbon from 1,4-phenylenediamine and formaldehyde via a hard-templating strategy, intended as an anode material for aqueous sodium-ion batteries. Our goal is to elucidate the evolution of the sodium-ion storage mechanism within carbon frameworks as a function of applied potential in assembled ASIBs, and to correlate these charge storage processes with their corresponding electrochemical signatures. Solid-state MAS NMR and temperature-dependent EPR studies were employed to provide detailed insights into the material’s structure-sodium storage relationship. Not simply that sodium is stored, but where it is stored within the hierarchical porous carbon architecture and how the storage sites evolve during sodiation. To explain this, we have adopted the only commonly used mechanistic terminology—adsorption, insertion, and intercalation to describe different modes of Na^+^ storage in carbon materials. Though these mechanistic definitions were originally developed from studies of nonaqueous electrolytes, they are still useful here, in our aqueous system, despite the fact that they do not operate through identical electrochemical processes and there is not an one-to-one correspondence between the aqueous and nonaqueous systems. To the best of our knowledge, this is the first mechanistic study using solid-state MAS NMR and EPR to investigate ASIBs. We believe that the broader contribution of this work lies in the development of an experimental framework for validating and refining sodium-storage mechanisms in emerging ASIBs, a class of systems for which mechanistic understanding remains considerably less developed than for their nonaqueous counterparts.

## 2 Experimental Section

### 2.1 Materials

Absolute ethanol reagent grade was obtained from Merck Millipore. Ammonia solution and 48% hydrofluoric acid (HF) were purchased from Fisher Chemicals. 98% Tetraethyl orthosilicate (TEOS), (3-aminopropyl) triethoxysilane (APTES), 1,4-phenylenediamine, 37 wt % formaldehyde, and sodium perchlorate (NaClO_4_) were purchased from Sigma-Aldrich. Super P conductive carbon and poly­(vinylidene fluoride) (PVdF) binder were purchased from GELON, and *N*-methyl-2-pyrrolidone (NMP) was purchased from Thermo Scientific. All materials were used as received.

### 2.2 Synthesis of Silica Template

Monodispersed spherical silica nanoparticles (S-SiO_2_) were prepared via a modified Stöber method.^20^ Initially, a mixture containing 150 mL of ethanol, 5 mL of deionized water, and 10 mL of ammonia was placed in a round-bottom (RB) flask and stirred at room temperature for 15 min. In a separate RB, 8 mL of tetraethyl orthosilicate (TEOS) was dissolved in 50 mL of ethanol to form a homogeneous solution. Then the TEOS solution was added dropwise to the initial mixture under continuous stirring. The resulting reaction mixture was maintained at 308 K for 8 h to promote particle growth. After completion of the reaction, the S-SiO_2_ nanoparticles were separated by centrifugation and washed multiple times with deionized water to remove residual reactants.

### 2.3 Synthesis of Conjugated Bowl-Shaped Hierarchical Porous Carbon (CBHPC)

One gram of S-SiO_2_ nanoparticles were dispersed in 100 mL of an ethanol-water mixture (1:1 v/v) and underwent ultrasonication for 4 h. Then 0.5 mL of (3-aminopropyl) triethoxysilane (APTES) was added dropwise, and the mixture was stirred for 18 h at pH 10 in order to induce silane coupling and introduce amine (−NH_2_) functionalities on the silica surface.^21^ This improves the reactivity of the material’s surface toward further modifications. In the next step, 1,4-phenylenediamine was introduced and stirred for 30 min until a clear pink solution was obtained. Then, 0.86 mL of formaldehyde (HCHO) was added, and the reaction mixture was stirred for an additional 12 h at 313 K. The resulting pinkish solid was thoroughly washed with a 1:1 ethanol-water mixture and dried in an oven at 323 K for 24 h. The completely dried material was then subjected to carbonization, during which the temperature was gradually increased to 1073 K at a heating rate of 278 K/min under an inert argon atmosphere. After carbonization, the sample was treated with 48% hydrofluoric acid (HF) to remove the silica template, followed by extensive washing with deionized water. The final product was designated as conjugated bowl-shaped hierarchical porous carbon (CBHPC). The synthetic route is illustrated in Scheme 1.

**Scheme 1:**
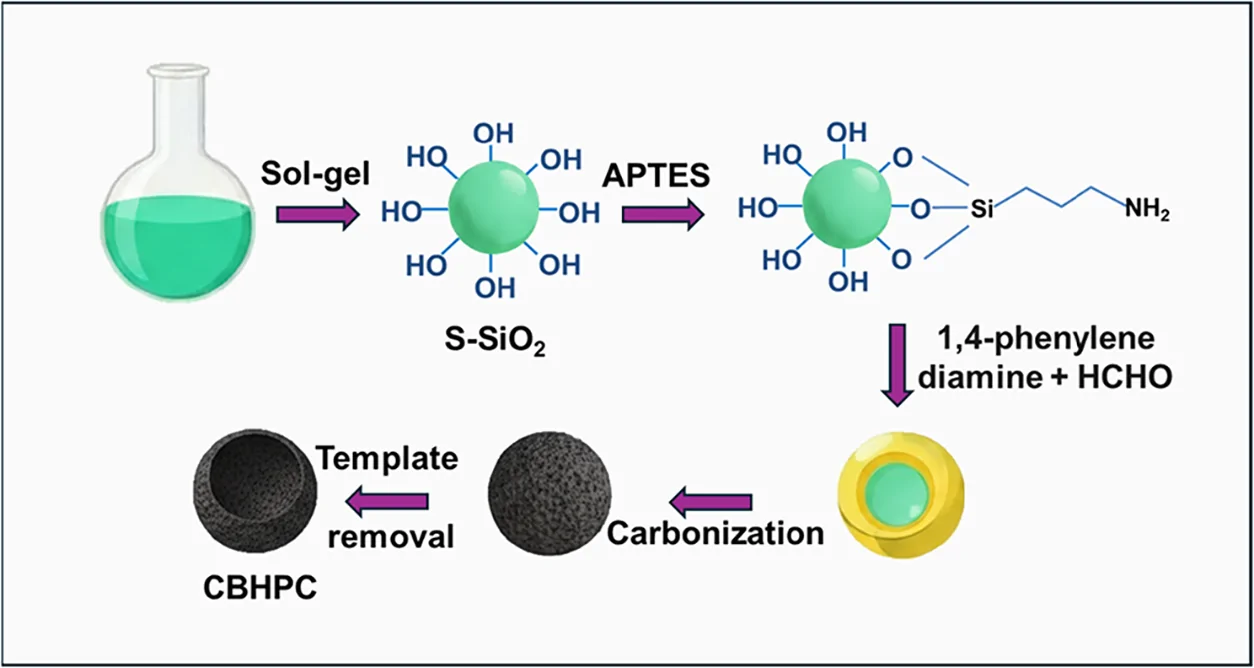
Schematic Illustration Depicting the Sequential Synthetic Pathway for the Fabrication of Conjugated Bowl-Shaped Hierarchical Porous Carbon (CBHPC)

The synthetic protocol of the present work is the amalgamation of the individual concepts, resulting in the formation of the conjugated bowl-shaped hierarchical porous carbon architecture.^22–24^

### 2.4 Electrode and Cell Fabrication

For the fabrication of the electrodes, CBHPC was mixed with a conductive agent (Super P) and a binder [poly­(vinylidene fluoride) (PVdF)] in a weight ratio of 8:1:1. *N*-methyl-2-pyrrolidone (NMP) was used as the solvent. The resulting slurry was coated onto the surface of titanium (Ti) foil. The as-fabricated electrodes were dried overnight at 338 K. Once the electrodes were completely dry, 6 mm diameter circular electrodes were punched out and used as the working electrodes. A carbon cloth of the same diameter (6 mm) was used as the counter electrode, and a silver wire served as the reference electrode. A 1 M aqueous solution of NaClO_4_ was used as the electrolyte. The entire cell assembly was performed using a Swagelok cell configuration to ensure good contact, and a stable electrochemical setup. Cyclic voltammetry (CV) and Galvanostatic charge–discharge (GCD) measurements were carried out using the Biologic VMP-3 potentiostat within the potential range of 0 to −0.9 V. In this work, a limited potential window has been chosen to maintain the stability of the aqueous electrolyte and suppress parasitic reactions such as the hydrogen evolution reaction. However, a practically useful full-cell voltage can be achieved via the correct choice of cathode, which will determine the full cell potential.

### 2.5 Sample Preparation for MAS NMR and EPR

CBHPC samples were electrochemically polarized for 90 min at a constant potential, in the range 0 to −0.9 V vs Ag/AgCl in a three-electrode Swagelok cell. After polarization, the material was divided into two batches: one batch was directly dried without washing, while the second batch was transferred to a Büchner funnel and gently washed with double-distilled water to remove excess electrolyte. Both sets of samples were then dried in a vacuum oven at 338 K overnight. The washed and unwashed samples were analyzed by MAS NMR and EPR.

### 2.6 Experimental Conditions of MAS NMR and EPR

Solid-state MAS NMR was performed on a Bruker AVANCE II 500 spectrometer using a commercial 4 mm double-resonance (^1^H, X) CP-MAS NMR probe, with a MAS frequency of 8 kHz. 90-aquire ^13^C and ^23^Na spectra were recorded, at frequencies of 125.77 and 132.31 MHz, respectively, using excitation pulse lengths of 4.58 us and 5.53 us, respectively. Experiments were also performed using a central transition-selective pulse, according to the equation *p*′ = *p*(1 + I)^−1/2^. However, these spectra did not indicate any qualitative difference from the standard 90° pulse experiments. The spectra were referenced to adamantane and 1 M NaCl_(aq)_, respectively. The recycle delays used for ^13^C and ^23^Na experiments were 120 and 0.5 s, respectively. For ^13^C measurements, *T*_1_ relaxation times were not explicitly measured, therefore a long relaxation delay was chosen to allow an overnight experiment with good SNR. The ^23^Na, *T*_1_ relaxation times were measured experimentally and found to be 0.72 ms. Based on the *T*_1_ value, substantially higher recycle delays (0.5 s) were chosen. The application of a relaxation delay greater than 5 × *T*_1_ ensures that the ^23^Na NMR spectra can be considered quantitative. To achieve optimum rotor stability, due to the small sample volume, ^13^C free spacers were used to fill the remaining rotor volume.

Continuous-wave (CW) electron paramagnetic resonance (EPR) spectra were recorded using a Bruker EMXplus spectrometer operating at X-band (9.5 GHz). The spectrometer is equipped with a Bruker high-sensitivity probehead. Each sample was placed inside a 4 mm EPR tube for the experiments. For low-temperature experiments (5 to 100 K), the sample was cooled using a ColdEdge/Bruker closed-cycle He refrigeration system. The resonator temperature was measured with a Lakeshore Cernox sensor. The EPR spectra were recorded at a center magnetic field of 3380 G with a sweep width of 1000 G. The microwave power was set to 0.6325 mW with an attenuation of 25 dB, and a field modulation amplitude of 1 G was applied. The number of scans varied across measurements; however, the spectrometer automatically normalizes signal intensity to account for differences in the number of scans between measurements.

The MAS NMR spectra were fitted using DMFit software.^25^ Fitting of the experimental EPR spectra was performed using EasySpin.^26^

### 2.7 Other Instrumentation

X-ray diffraction (XRD) patterns of CBHPC were recorded using a Bruker D8 Advance diffractometer equipped with Cu Kα radiation (λ = 1.5406 Å). Raman spectra were collected on an InVia Confocal Raman Microscope (Renishaw). Prior to analysis, the sample was dispersed in ethanol, drop-cast onto a silica wafer, and dried under ambient conditions. Nitrogen sorption isotherms were measured at 77 K using a Quantachrome Nova 1200e surface area and pore size analyzer. Before measurement**s**, the samples were degassed under vacuum at 393 K for 8–12 h. The specific surface areas were calculated from the adsorption isotherms using the Brunauer–Emmett–Teller (BET) method, and the pore size distribution profiles were determined using the nonlocal density functional theory (NLDFT) model. The High-resolution scanning electron microscopy (SEM) (Apreo 2S, Thermo Fisher Scientific) was employed to investigate the morphology of the material. Additionally, the detailed microstructure was examined using an aberration probe-corrected scanning transmission electron microscope (TEM) (Themis Z G3, Thermo Fisher Scientific, formerly FEI). X-ray photoelectron spectroscopy (XPS) measurements were performed using an Axis Supra spectrometer (Kratos) with monochromatic Al Kα radiation as the X-ray source to determine the elemental composition and chemical states of the material.

## 3 Results and Discussion

Monodisperse S-SiO_2_ nanoparticles were synthesized via a modified Stöber method and subsequently functionalized with a silane coupling agent (APTES). The surface functional groups introduced by the silane coupling agent effectively bridge the inorganic silica core with the organic monomer.^21^ This facilitated the uniform in situ surface polymerization of 1,4-phenylenediamine and formaldehyde, yielding a well-defined core–shell structure comprising a silica core and a poly­(1,4-phenylenediamine-formaldehyde) shell. Carbonization and template removal yielded CBHPC.

### 3.1 Characterization of the Pristine Carbon

#### 3.1.1 Characterization of the Pristine Carbon via Spectroscopic and Microscopic Techniques

The pristine CBHPC was characterized via several spectral and microscopy techniques. The high-angle powder XRD spectrum of the CBHPC in Figure 1a shows its amorphous features. A strong peak appears at a 2θ value of 24.4°, corresponding to the (002) plane, and another small peak at 43.7° is observed, which corresponds to the presence of the (100) lattice plane in the samples. The (002) reflection corresponds to the interlayer spacing between graphitic planes. In ideal graphite, this peak appears at ∼26.5° (2θ), corresponding to an interlayer spacing of 0.335 nm. A shift to lower angles (∼24°) implies a larger interlayer distance (∼0.37 nm), which is common in turbostratic or disordered carbon. The turbostratic layer is often observed in porous carbon due to defects, heteroatom doping (e.g., N, O), or partial graphitization.^27^ However, a minimum interlayer spacing of ∼0.37 nm, corresponding to the (200) plane, is advantageous for sodium storage.^28^ The relatively high intensity of the (002) reflection further indicates a significant degree of graphitic domain formation within the hierarchical porous carbon structure.^29^

**Figure 1:**
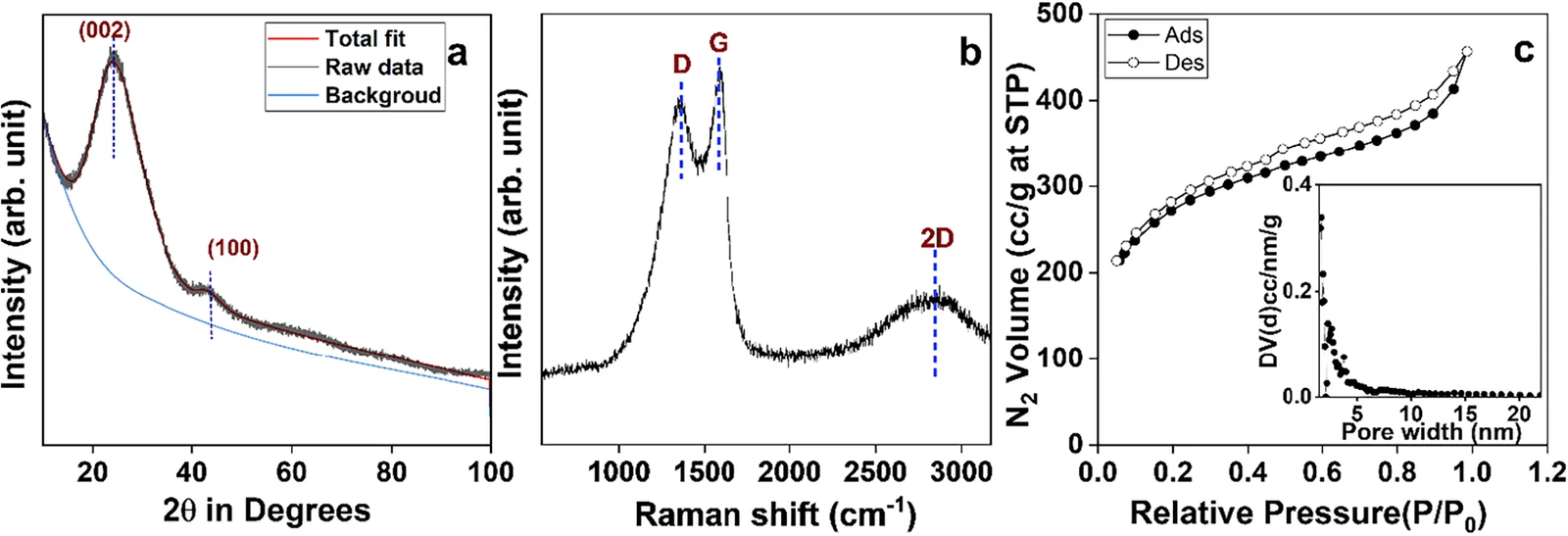
(a) Powder X-ray diffraction (XRD) spectra, (b) Raman spectra, and (c) Nitrogen adsorption/desorption isotherms of CBHPC. The *inset* in (c) illustrates the pore-size distribution of CBHPC.

The Raman spectrum of the CBHPC (see Figure 1b) features three distinct characteristic peaks: D, G, and 2D bands. The D-band at 1352.7 cm^–1^ corresponds to structural defects and disordered regions in the graphitic carbon framework. The appearance of the G band at 1591.6 cm^–1^ is attributed to the E_2g_ vibrational mode of sp^2^-hybridized carbon atoms within the graphite plane. The presence of a 2D-band at 2680 cm^–1^, a signature feature of graphitic carbon materials, further confirms the graphitic nature of CBHPC.^30^ The intensity ratio between D- and G-band (*I*_D_/*I*_G_) reflects the degree of graphitization; the higher the value, the less graphitization. CBHPC exhibited an *I*_D_/*I*_G_ value of 0.91, indicating the presence of abundant defects in the graphitic domains.^31^

Nitrogen adsorption-desorption measurements were performed on CBHPC to determine its surface area and pore-size distribution. The resulting isotherms (Figure 1c) exhibit a type-IV pattern with small hysteresis loops in the relative pressure range of approximately 0.5–1.0, indicating the presence of large mesopores in the carbonized material.^32^ The material shows a high specific surface area of 917.5 m^2^/g. The *inset* of Figure 1c displays the NLDFT pore size distribution of CBHPC, revealing multiple pore size distributions with pore widths of 1.7, 1.8, 2.3, 2.6, and 3.8 nm, suggesting the presence of hierarchical porosity within the structure.

SEM images of CBHPC, shown in Figure 2a, indicate well-defined bowl-shaped particles with an average diameter of approximately 320 nm. This morphology suggests the formation of a large internal cavity within the carbon shell, formed upon the removal of the silica template during the carbonization stage. The *inset* in Figure 2a shows a closer view of the CBHPC particles, where the surface appears relatively smooth. The particles adopt a clear bowl-like shape, similar to deflated balloons. Once the spherical SiO_2_ templates are removed (Figure S1), the porous carbon framework no longer has enough structural support to retain a hollow spherical form. Instead, the shell collapses inward, producing the bowl-shaped morphology.

**Figure 2:**
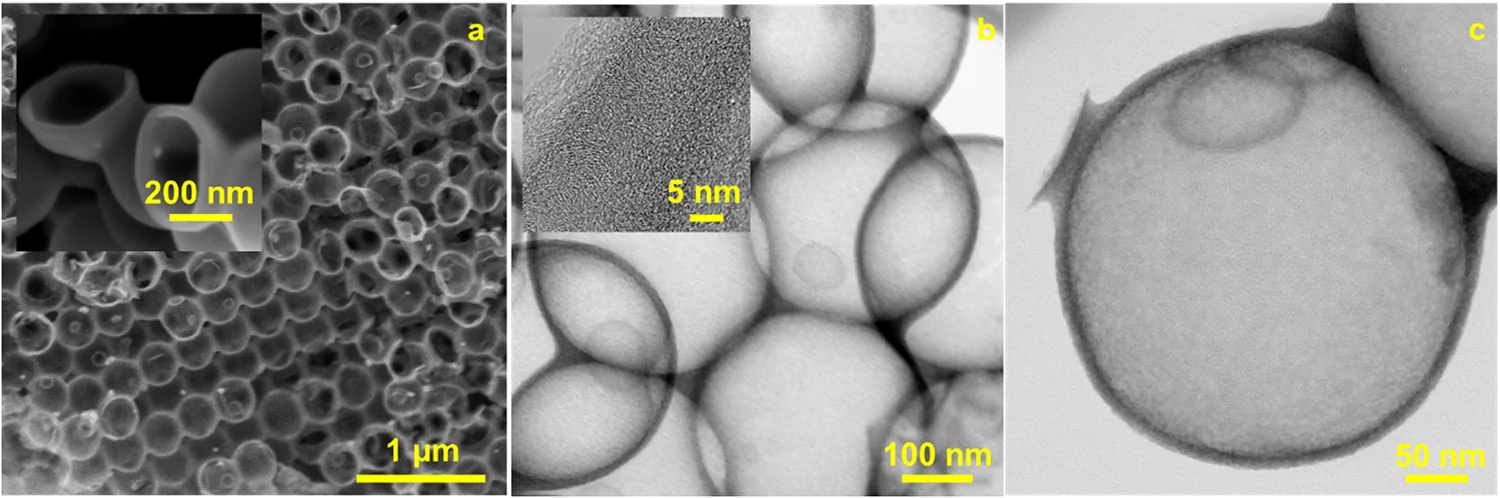
(a) SEM image of CBHPC; the *inset* shows a magnified view of the particles, (b) TEM image of CBHPC; the *inset* highlights the graphene layers, and (c) high-magnification TEM image showing detailed morphology of CBHPC particles.

Next, we performed TEM on the CBHPC particles, the results of which are shown in Figure 2b,2c. Figure 2b shows the hollow-shaped particle of the CBHPC particles. Figure 2c displays a magnified view of a single particle with a wall thickness of 15.4 nm. The *inset* of Figure 2b displays high-resolution TEM images that reveal the presence of graphene layers, as thin sheet-like structures with lattice fringes. Indeed, these fringes show parallel spacing of about 0.34 nm, which corresponds to the interlayer distance between stacked graphene sheets in graphitic carbon.^33^ This observation is further supported by XRD analysis (Figure 1a), which shows a corresponding interlayer spacing of ∼0.37 nm. Unlike crystalline graphite, these layers are not perfectly stacked, leading to a highly disordered structure. The curvature and misalignment of the graphene layer create abundant, randomly distributed pores throughout the framework, contributing to the material’s pronounced hierarchical porosity.^34^ In addition, the presence of structural defects and the high degree of disorder facilitates rapid sodium-ion transport, thereby improving battery performance in ASIBs.

XPS studies were carried out on the CBHPC to determine its surface elemental compositions and chemical states. The survey spectrum shown in Figure 3a, CBHPC, reveals the presence of C, N, and O atoms within the carbon framework. The elemental contents are given in the *inset*, indicating the presence of C, N, and O are 89.25%, 6.6%, and 3.8%, respectively. A negligible amount of F (0.35%) is also detected, likely originating from residual impurities introduced during HF etching. In Figure S2a, we present the deconvoluted C 1s spectra of all samples, which exhibit a peak at approximately 285.0 eV, corresponding to the presence of C–C and C–H bonds in the carbon framework, whereas a peak at 286.3 eV appears for C–O bonds.^35^ The O 1s spectrum is deconvoluted into two peaks at 530.6 eV, 532.6 eV, indicating the presence of C=O, C–O/C–O–H functionality (Figure S2b). In Figure 3b, the XPS spectrum of N 1s is deconvoluted into three peaks at 398.0, 400.5, and 402.6 eV, indicating the presence of pyridinic-N, pyrrolic-N, and graphitic-N, respectively.^22^ The peak intensity of pyrrolic-N is higher than that of pyrrolic-N. Substitution of carbon atoms with nitrogen in the carbon framework of CBHPC facilitates the formation of C–C and C–N defect sites.

**Figure 3:**
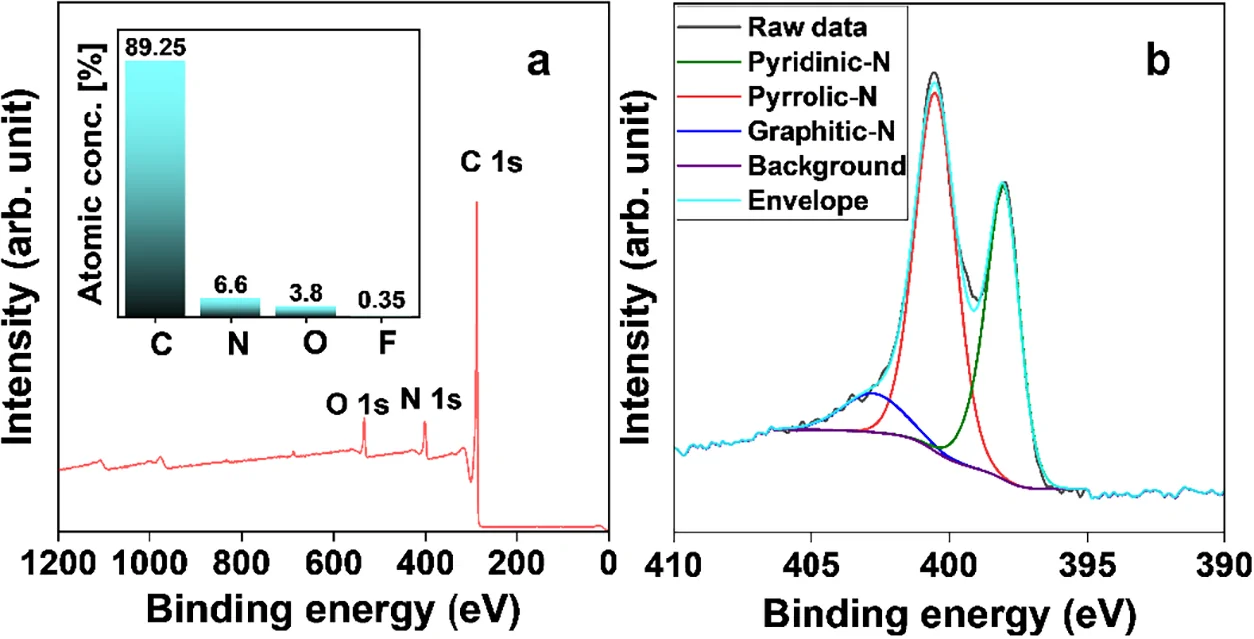
(a) XPS survey spectrum of CBHPC. The *inset* shows the atomic percentage composition. (b) High-resolution XPS spectrum of the N 1s core level of CBHPC.

#### 3.1.2 Characterization of the Pristine Carbon via Magnetic Resonance Techniques

To further study the structural transformations occurring in CBHPC, we performed CW x-band EPR and MAS NMR experiments. ^13^C NMR solid-state MAS NMR was conducted on pristine CBHPC to understand the carbon framework at the molecular level (Figure S3). The ^13^C NMR spectrum of CBHPC shows a single peak at 111.7 ppm, which is the result of the presence of sp^2^ hybridized –C=C– carbons inside the carbon framework.^36^ Imai et al. reported that a chemical shift at ∼116 ppm corresponds to a graphite-like core structure. Therefore, the observed peak at 111.7 ppm in our work can also be attributed to a graphite-like core structure within the carbon framework. This assignment is in good agreement with the TEM results.^37^

Room-temperature CW-EPR was performed in order to study the intrinsic paramagnetic centers and the electronic structure of carbon materials under ambient, application-relevant conditions. Complementary temperature-dependent CW-EPR analysis is employed to understand the thermal evolution of paramagnetic centers and unpaired electron dynamics in carbon materials.

Figure 4a shows the EPR spectrum of pristine CBHPC recorded at room temperature, exhibiting a single resonance peak at *g* ≈ 2.0040. This signal arises from unpaired electrons associated with structural defects within the carbon framework.^38^ This sharp EPR spectrum is further fitted with two Dysonian line shapes (Figure 4a), consisting of Line-1 (*g* = 2.0055) and Line-2 centered at *g* = 2.0040. The observed g-values further confirm carbon-centered defects in the hierarchical CBHPC,^39^ attributed to graphene edges, voids in open micropores, vacancies within the graphene sheets, and dangling bonds terminated with oxygen or nitrogen groups (as indicated by XPS).^40^ The appearance of two EPR fitting components is governed by the synthesis temperature of CBHPC. When carbon is synthesized above 973 K, the thermal treatment influences its magnetic and electronic properties, causing the EPR spectrum to evolve from a single line to two components with distinct line widths, due to carbonization and graphitization, one of localized electrons and one of more mobile electrons.^18^

**Figure 4:**
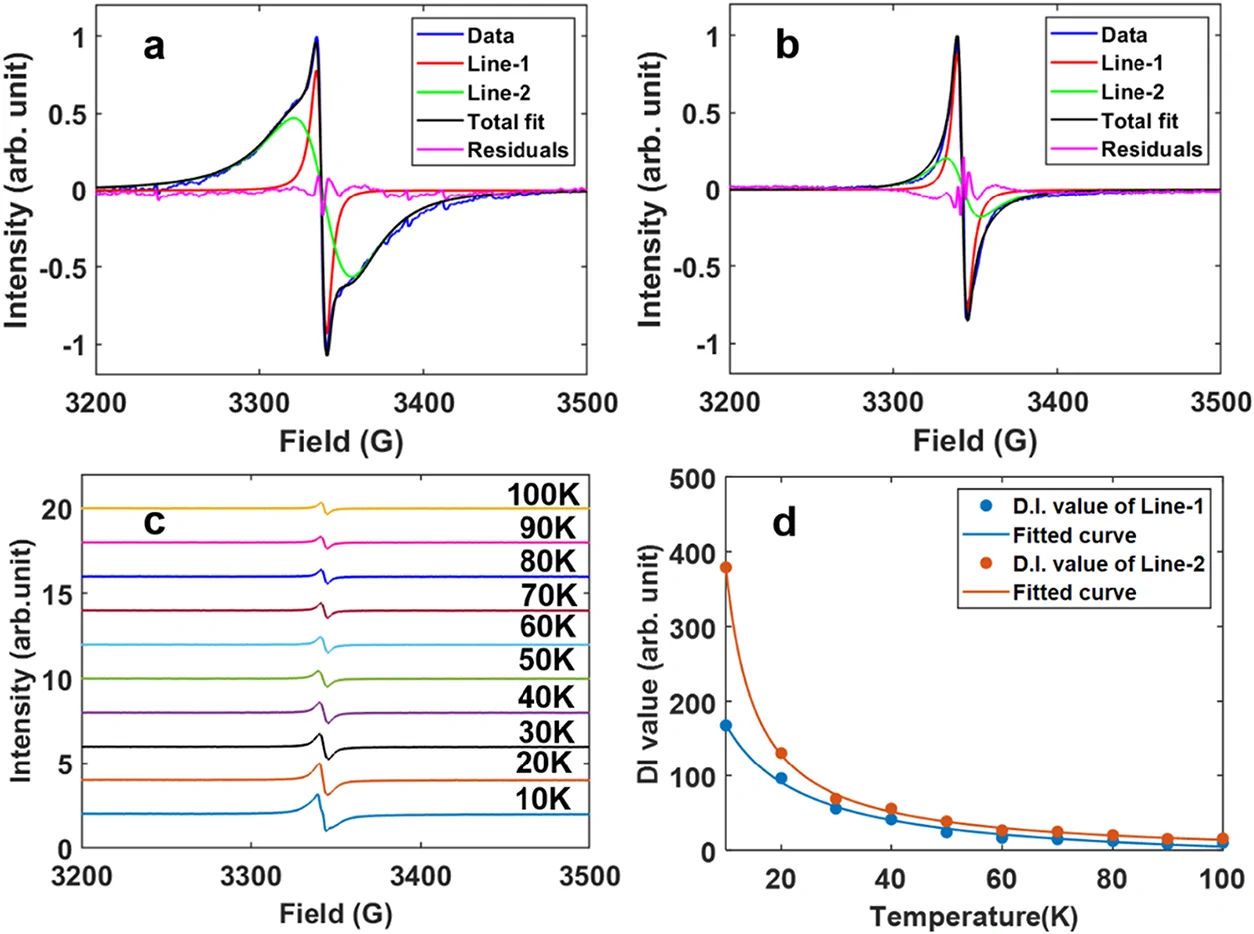
EPR characterization of CBHPC: (a) EPR spectrum measured at room temperature (blue); (b) EPR spectrum measured at 10 K (blue). In (a, b), both spectra are overlaid with the corresponding fits for Line-1 (red) and Line-2 (green), their sum (i.e., the total fit) (black), and the residuals (magenta). (c) EPR spectra recorded at different temperatures, and (d) double integration value of Line-1 and Line-2, plotted as a function of temperature. The solid line overlaid on the spectra denotes the Curie–Weiss fitting.

The Dysonian line shape observed in fitting the EPR signal of pristine carbon indicates the presence of some metallic-like behavior in CBHPC. The extent of the metallic/conductive behavior can be characterized using the Dysonian asymmetry value, A/B ratio corresponding to the low-field and high-field peak amplitudes.^41^ The larger the value of the asymmetry, the more metallic and conductive the sample is.^42^ Here, we have calculated the Dysonian asymmetry, and the value is 0.8339 at room temperature. In the variable temperature experiments, discussed below, this parameter increases at lower temperatures, but attains a maximum at 1.1287 even at 10 K (see Table S1). This relatively low A/B ratio suggests a significant contribution from localized paramagnetic centers consistent with the presence of partially delocalized electrons in CBHPC.

Figure 4b represents the EPR spectrum of CBHPC at 10 K temperature fitted with two lines: (Line-1 at *g* = 2.0037, Δ*H*_pp_ = 6 G and Line-2 at *g* = 2.0037, Δ*H*_pp_ = 20.9 G). Wang et al. and other researchers have similarly attributed EPR spectra exhibiting two components with comparable peak-to-peak line widths (Δ*H*_pp_) to distinct carbon-centered defect environments. In this framework, the narrow line corresponds to localized σ-type dangling bonds/defects, while the broader component may be associated with localized π electrons originating from aromatic domains.^43–45^ It is known that π electrons become localized when extended conjugation is disrupted by defects, finite aromatic domain size, or structural disorder, particularly at low temperatures where thermally assisted delocalization is suppressed.^19^ Figure 4c presents the temperature-dependent spectra of CBHPC recorded between 10 and 100 K, using the two-component fitting described above. Both the signal intensity and line width of each of the components (Table S1) progressively decrease with increasing temperature. The decrease in the EPR Δ*H*_pp_ with increasing temperature is consistent with motional narrowing due to thermally activated spin motion averaging out local field inhomogeneities, as observed in EPR studies of organic semiconductors and carbon systems in the literature.^46^ At low temperatures, the intensity of the EPR signal is directly proportional to the magnetic susceptibility of the material. As the temperature increases, thermal motions randomize the spin populations, resulting in a reduction of the net magnetic susceptibility and, consequently, a decrease in EPR signal intensity as well.^47^ All the fitting parameters are given in Table S1.

To fully characterize the change in signal intensity as a function of temperature, we have performed double integration (DI) of the first-derivative EPR spectra. The DI intensity reflects the spin susceptibility of the system. The temperature-dependent spin susceptibility, χ­(*T*), can be described using a combined Curie–Weiss and Pauli paramagnetic model

1 $$\chi \left(T\right)=\chi_{\mathrm{Pauli}}+C/\left(T-T_{0}\right)$$

where, χ_Pauli_ is the temperature-independent contribution arising from delocalized conduction electrons, characteristic of Pauli paramagnetism in graphitic domains.^19^ *C* is Curie constant; *T* is the temperature in Kelvin, *T*_0_ is the Curie–Weiss temperature in Kelvin, and *C*/(*T* – *T*_0_) represents the contribution from localized spins following Curie–Weiss behavior, associated with carbon defects, dangling bonds, or edge functionalities. Figure 4d shows that, in CBHPC, both Line-1 and Line-2 follow a Curie–Weiss-type temperature dependence. As the temperature increases, the double integral (DI) values gradually decrease according to eq 1. This observation is consistent with the Curie–Weiss behavior of localized electrons.^16^ These results confirm that the observed EPR signals originate from localized carbon-centered defects, including σ-type dangling bonds and localized π-electron states. The unpaired electrons are, most likely, situated either along the edges of graphene sheets, within micropores, and vacancy sites in the carbon lattice of the hierarchical carbon framework of the CBHPC. This behavior also suggests that magnetism arises from defects rather than from band structure, consistent with disordered or porous carbon frameworks.^48^ The fitting parameters are provided in Table S2.

### 3.2 Sodium Storage: Mechanism

#### 3.2.1 Sodium Storage: Electrochemical Performance of CBHPC

First, we performed cyclic voltammetry on samples in half-cells, within a voltage window of 0.0 to −0.9 V. The CV curve, shown in Figure 5a, exhibits quasi-rectangular patterns without any noticeable redox behavior, indicating that both electrical double-layer (EDLCs) and diffusion-controlled processes are occurring during the charge storage process.^22^ At lower scan rates, such as 0.1 mV/s (plotted again in Figure 5b), the CV curves show two weak humps, one around −0.1 to −0.2 V, and another in between −0.5 to −0.7 V. These redox features suggest reversible Faradaic processes, which can be attributed to Na^+^ interactions with defect sites, edge functionalities, and oxygen-containing surface groups in the porous carbon framework.

**Figure 5:**
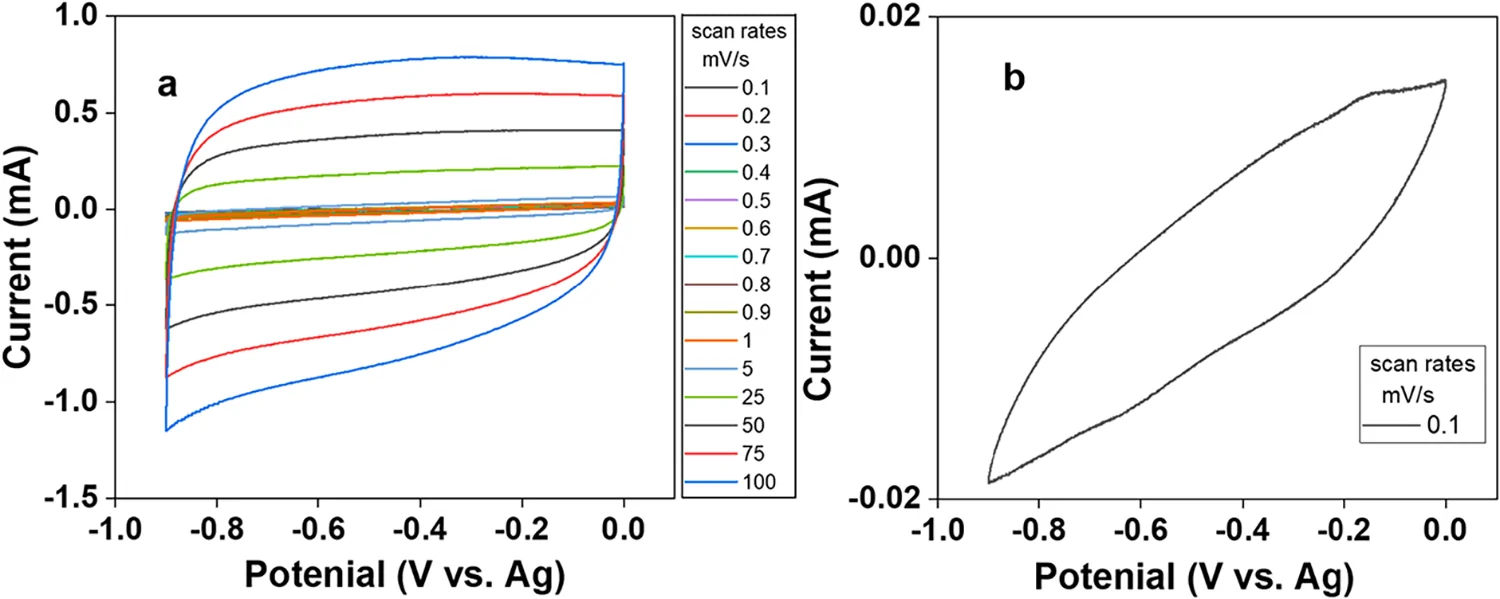
Represents (a) Cyclic voltammogram of CBHPC scan rates ranging from 0.1 to 100 mV/s, (b) Enlarged CVs at lower scan rates (0.1–1 mV/s).

Adsorption and insertion are two of the most important sodium storage mechanisms in porous carbon. Kinetic analysis based on CV curves is an effective approach to elucidate the rate-determining steps involved in the sodium storage process. The relationship between the measured peak current (*i*_p_) and the scan rate (*v*) follows a power law, expressed as *i*_p_ = *av^b^*, where the *b* value can be determined from the slope of the log­(*i*_*p*_)–log­(*v*) plots. Specifically, a *b* value of 0.5 corresponds to a diffusion-controlled process, whereas a value of 1.0 indicates a surface-controlled (capacitive) process.^49^ Figure S4a displays the CV curves of CBHPC at various scan rates ranging from 0.1 to 100 mV/s. The log­(*i*_p_) values exhibit a linear relationship with log­(*v*). At a potential of −0.2 V, the fitted *b* value is 0.62, which is quite close to 0.5, indicating that the sodium storage process at low potential is predominantly diffusion-controlled. At higher scan rates (5 to 100 mV/s, Figure S4b), however, the fitted *b* value approaches 0.89, suggesting the coexistence of two overlapping processes, namely chemical adsorption and sodium insertion. Moreover, Dunn’s method^50^ was employed to calculate the capacitive and diffusion-controlled contributions from the CV curves. *i*(*V*) *= k*_1_ *v* *+ k*_2_ *v* ^0.5^, where *k*_1_ *v* and *k*_2_ *v* ^0.5^ represent the capacitive contributions and diffusion-controlled contribution, respectively. *v* is the scan rate, and *k*_1_ and *k*_2_ are the partial contributions of capacitive contributions and diffusion-controlled contributions.^51^ Figure S5 reveals that the diffusion-controlled contribution at scan rates of 0.5, 1.0, and 5.0 mV/s is approximately 70%, 66%, and 18%, respectively, and a gradual enhancement of the scan rate results in an increase to the capacitive contribution. Thus, at a low scan rate, the diffusion-controlled process predominates.

GCD measurements were performed at a current density of 0.1 A/g, as shown in Figure 6a. In this voltage profile, the charge and discharge processes correspond to the insertion and extraction of sodium ions, as the hierarchical porous carbon serves as the negative electrode in a full-cell setup. The pattern of the GCD curves in Figure 6a,6b deviates from the ideal triangular shape. They exhibit both sloping and plateau-like regions, reflecting combined capacitive and diffusion-controlled storage.^52^ The CBHPC delivers a specific capacity exceeding 250 mAh/g at 0.1 A/g. This high specific capacity of the CBHPC electrode, primarily arises from its unique hierarchical nanostructure, which maximizes the utilization of electrochemically active material and shortens Na^+^ diffusion pathways.^53^ Its large specific surface area (917.5 m^2^/g), which provides abundant active sites for Na^+^ adsorption. The porosity of the carbon framework and the presence of interconnected particles ensure efficient electrolyte penetration and rapid ion accessibility. The ultrathin pore walls (∼15.4 nm) shorten diffusion pathways, thereby enhancing reaction kinetics and improving the overall electrochemical performance.^54^ Moreover, the bowl-shaped particle morphology exposes a larger electrolyte-accessible surface than conventional spherical particles, improving Na^+^ access to the pore network and facilitating faster ion transport into the electrode.^55^ The contribution of these structural features enables the CBHPC electrode to achieve a high specific capacity in an aqueous electrolyte.

**Figure 6:**
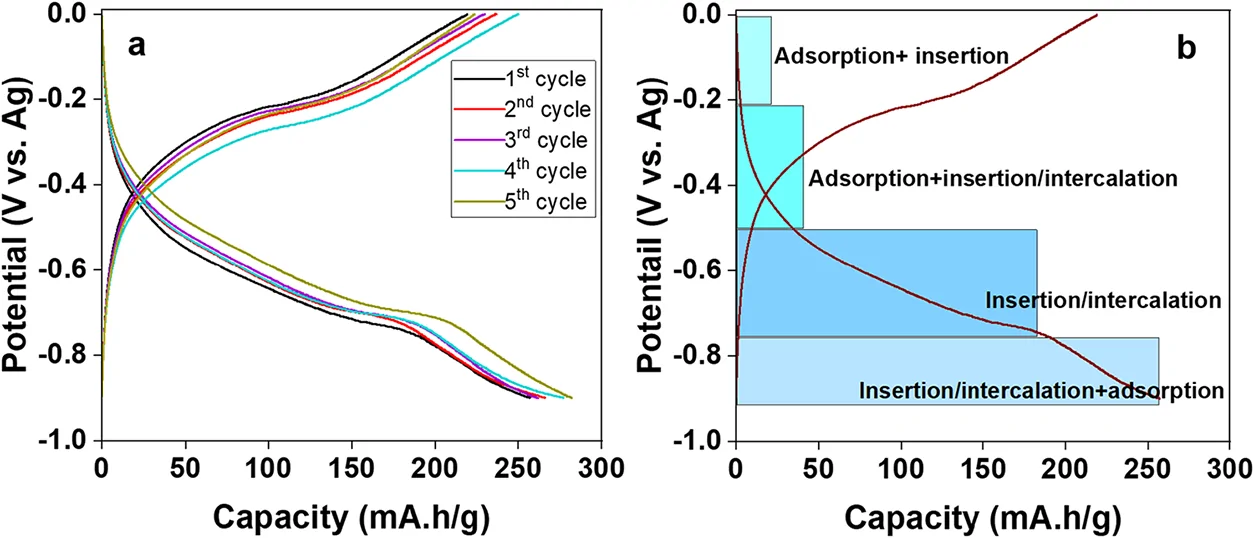
(a) The galvanostatic charge–discharge (GCD) measurements of CBHPC at 0.1 A/g current density. (b) Enlarged GCD for the 1st cycle, the slopes of the curve suggest different stages of adsorption/insertion/intercalation.

The GCD profile of the porous carbon anode shows an initial steep sloping region up to −0.2 V, which can be associated with the formation of a solid-electrolyte interphase (SEI). Upon contact between the aqueous electrolyte and the carbon surface of CBHPC, thermodynamic instability at the interface drives irreversible electrolyte decomposition, leading to the formation of a SEI,^56^ giving rise to an early insertion/intercalation-adsorption process that governs the initial charge–discharge response. Further decrease of the negative potential up to −0.5 V leads to the formation of a mixed charge storage mechanism, combining EDLC and insertion/intercalation, corresponding to the surface adsorption of sodium ions at defect sites and graphene edges.^57^ As the potential becomes more negative, the slope transitions into a distinct plateau around −0.7 V, indicative of Na^+^ insertion/intercalation into graphitic domains and filling of nanopores. The nanopores, formed as the voids between randomly stacked graphitic domains, can accommodate Na^+^ ions and are responsible for the low-potential plateau capacity that governs the charge–discharge behavior.^58^

At even more negative potentials (−0.9 V), the profile exhibits a sloping behavior, reflecting additional adsorption or insertion/intercalation at less-accessible defect sites and/or partially aggregated Na^+^ in the pore walls. The graphitic domain, formed by the random stacking of few-layer graphene sheets, is generally associated with Na^+^ insertion/intercalation into stacked graphene or adsorption at defective graphitic surfaces, processes that contribute to the sloping capacity.^59^ The pore filling is the occupation of all or nearly all the available space within the nanopores. Considering the strong Coulombic repulsion among positively charged sodium ions in the narrow micro- to mesopores, sodium filling in these nanopores is proposed to occur primarily in atomic or neutral forms.^60^ Another theory focuses on the sloping potential region, which is associated not only with the storage of ionic sodium at sites near localized electron spin resonance centers, such as dangling bonds or terminated free radicals, but also with the insertion of sodium ions into the interlayer spaces between carbon layers via charge transfer from sodium to carbon.^61^ The charge-storage mechanisms predicted from electrochemical analysis are summarized in Figure 6b and Column 1 of Table 1. In Figure S6, variations in Coulombic efficiency (CE) across various current densities are plotted; at a low current density of 0.1 A/g, it is below 85%. However, the CE progressively increases with increasing current density and reaches ∼100% at moderate to high current densities. The reason is that longer charge/discharge times for very low current density allow more irreversible side reactions, such as electrolyte decomposition, self-discharge, ion trapping, and surface reactions. In contrast, at higher current densities, these parasitic reactions contribute a much smaller fraction of the total charge, leading to a higher CE.^62^

**Table 1:** Correlation of NMR, EPR Results with Galvanostatic Charge–Discharge Data

Polarization potential	Charge storage mechanism predicted by electrochemical analysis	Charge storage mechanism predicted by ^23^Na-MAS NMR analysis	Charge storage mechanism predicted by EPR analysis
0 V	—	No Na^+^ insertion	—
–0.2 V	Early insertion/intercalation-adsorption	Adsorption and intercalation	Adsorption
–0.5 V	EDLC and ion insertion	EDLC and ion insertion	Ion adsorption-driven EDLC
–0.7 V	Insertion/intercalation and pore filling	Ion insertion and pore filling	Insertion/intercalation
–0.9 V	Insertion/intercalation and adsorption	Intercalation	Intercalation

From the electrochemical analysis alone, it is not possible to unambiguously assign the contributions of different charge storage mechanisms. This makes it difficult to distinguish whether adsorption or insertion/intercalation mechanisms predominate. Similarly, the location of ion insertion, whether within nanopores or between graphene layers, cannot be determined from electrochemical data alone. To gain further insight into the storage mechanisms and the local environment of the ions, complementary analyses were conducted via solid-state MAS NMR and EPR.

#### 3.2.2 Sodium Storage: NMR Analysis

The aim of this work was to determine whether the ions are adsorbed solely on the external surface or also within the interlayer pores. The unwashed samples exhibited a strong resonance peak at −6.5 ppm, corresponding to residual electrolyte (Figure S7).^63^ To exclude contributions from residual electrolyte, direct detection ^23^Na-MAS NMR experiments were performed with washed polarized CHPBC electrodes.

The washed samples displayed ^23^Na-MAS NMR signals at different chemical shifts depending on the applied polarization potential. In the literature, resonances observed between +30 ppm and −60 ppm are attributed to inserted sodium ions occupying various nanoporous sites within the hierarchical carbon structure (see Figure 7).^64^ Three distinct ^23^Na NMR environments are commonly observed: (i) Na^+^ ions appearing in void spaces between graphene layers around 7–12 ppm, (ii) monodispersed ions near −2.7 ppm, and (iii) aggregated Na^+^ ions in the nanopores showing resonances in the range of −12 to −23 ppm.^65,66^ The relative intensity plots corresponding to these spectra are included in the Supporting Information section (Figure S8). At a polarization potential of 0 V, no peak is observed in the ^23^Na-MAS NMR spectrum, indicating the complete deinsertion of Na^+^ ions. When the sample was polarized to −0.2 V, two broad peaks appeared, deconvoluted into two distinct signals at 12.6 ppm and −2.8 ppm, corresponding to Na^+^ ions in graphitic-like domains^67^ and monodispersed Na^+^ ions,^66^ respectively, indicating that both Na^+^ adsorption in/on defect sites and Na^+^ intercalation between graphene layers control the charge-storage mechanism. At −0.5 V, these same sodium species are seen, though slightly shifted to 8.6 ppm,^66^ −2.7 ppm, and an additional peak appears at −13.1 ppm, attributed to surface-aggregated Na^+^ ions.^68^ The presence of aggregated Na^+^ ions together with monodispersed Na^+^ ions indicates that both Na^+^ insertion into surface nanopores and Na^+^ adsorption at defect sites are active charge-storage mechanisms; however, the relatively strong signal corresponding to monodispersed Na^+^ suggests that adsorption-dominated EDLC behavior is the predominant storage mechanism in this potential range. At −0.7 V, peaks are seen at −10.07 ppm, and −19.9 ppm, corresponding to the interaction of Na^+^ ions with C–O/C–O–H functionalities of the hierarchical carbon center,^63^ and aggregated Na^+^ ions, respectively. The peak at −7.1 ppm is a result of traces of sodium electrolyte, which were not completely removed after washing. Within this potential range, Na^+^ ions appear mainly in aggregated, interacting states. This suggests a redox-driven charge-transfer process, which may proceed via intercalation or insertion. However, no clear redox peaks associated with Na^+^ in graphitic-like domains are observed. This suggests that Na^+^ insertion into nanopores is the dominant charge-storage process. At −0.9 V, the intense narrow peak around 7.6 ppm indicates that the sodiation process is dominated by Na^+^ insertion into the void spaces between graphene layers.^64^ The peak at −21.6 ppm also indicates the presence of Na^+^ ions inside the nanopores in an aggregated state, where it is expected to have restricted mobility. Thus, at less negative potentials, Na^+^ ions are less able to access the carbon framework of CBHPC. Under these conditions, the ions are mostly dispersed, giving rise to peaks at −2.8 ppm (at −0.2 V) and −2.7 ppm (at −0.5 V). Upon further negative polarization, the Na^+^ ions begin to insert into the structure and start to aggregate, allowing deeper penetration into the carbon framework. This shift marks a transition from surface-driven adsorption to storage dominated by intercalation within the porous carbon matrix upon further polarization.

**Figure 7:**
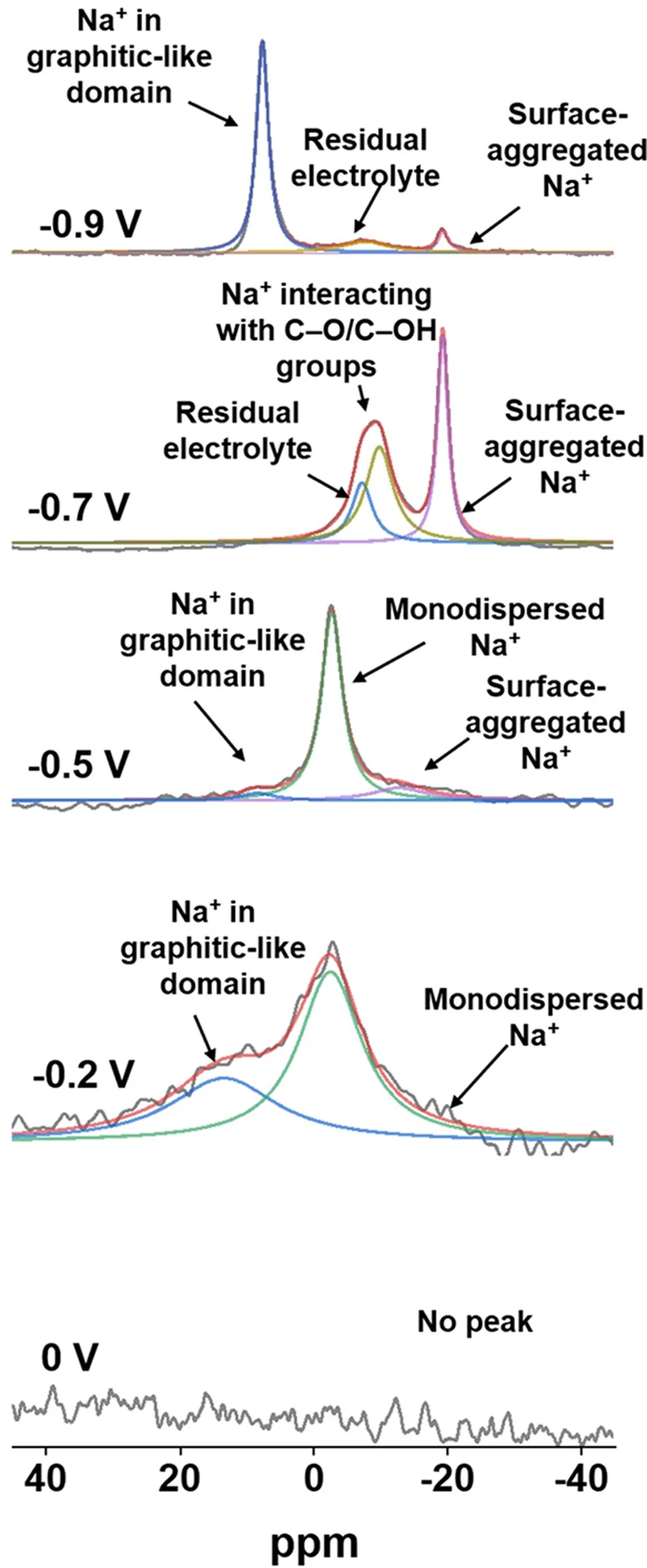
Normalized direct-detection ^23^Na NMR spectra of CBHPC samples charged at different potentials, after thoroughly washing to remove residual electrolyte. The experimental data (black) is overlaid with the fits (red) which are made up of several NMR lines (colored lines).

#### 3.2.3 Sodium Storage: EPR Analysis

To gain deeper insight into the subtle structural and electronic changes occurring within the CBHPC carbon framework under different applied potentials (0 V, −0.2 V, −0.5 V, −0.7 V, and −0.9 V), EPR measurements were conducted at room temperature and at cryogenic temperatures (10–100 K).

In Figure 8a, we show the EPR spectrum of the −0.9 V CBHPC sample at room temperature, as an example of a typical spectrum overlaid with its fit. No significant differences are observed in the EPR spectra of the polarized CBHPC samples. The spectra at other potentials and their fits are plotted in the Figure S9. The EPR spectra recorded at all polarized potentials display a consistent pattern of two overlapping peaks, which are fitted with two Dysonian line shapes, with a low asymmetry value A/B ratio ranging from 0.7234 to 1.1061 (Tables S3–S7), and follow a similar pattern for the fitting of the EPR spectra performed on the pristine CBHPC sample shown above (Figure 4). At room temperature, Line-1 exhibits a *g*-value in the range of 2.0056–2.0057, and line width (Δ*H*_pp_) values varying from 2.4 to 2.8 G (see Tables S3–S7). Line-2, at RT, indicates g-values in the range of 2.0054–2.006, with Δ*H*_pp_ values varying from 7.2 to 12.1 G (see Tables S3–S7) for all polarization states.

**Figure 8:**
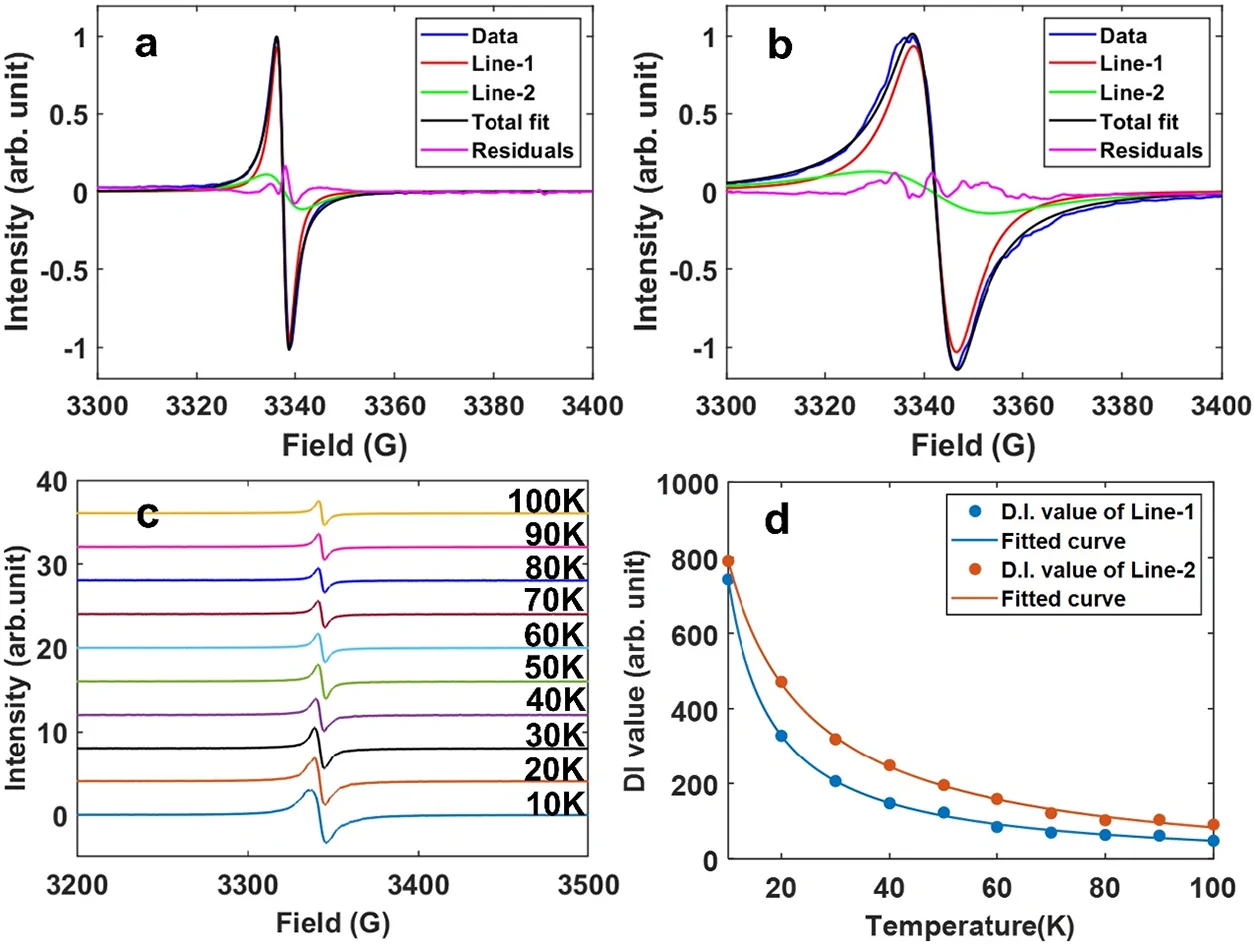
EPR spectrum of CBHPC polarized at −0.9 V: (a) EPR spectrum measured at room temperature (blue); (b) EPR spectrum measured at 10 K (blue). In (a, b), both spectra are overlaid with the corresponding fits for Line-1 (red) and Line-2 (green), their sum (i.e., the total fit) (black), and the residuals (magenta). (c) EPR spectra recorded at different temperatures, and (d) the double integration values of Line-1 (blue) and Line-2 (red) plotted as a function of temperature for samples polarized at −0.9 V. The experimental data are overlaid with a fit using the Curie–Weiss equation (solid lines).

Next, we discuss the results of the EPR measurements measured at 10 −100 K. Figure 8b displays the EPR spectrum of the CBHPC sample polarized at −0.9 V, measured at 10 K. Spectra of the samples polarized at 0 V, −0.2 V, −0.5 V, and −0.7 V measured at 10 K are shown in Figure S10. All spectra are well fitted with Dysonian line shapes. The g-values for Line-1 vary in the range of 2.0031–2.0057, while those for Line-2 vary in the range of 2.0026–2.0057 across all polarized CBHPC frameworks at 10 K. These results indicate that, similar to pristine CBHPC, the polarized CBHPC frameworks also exhibit the coexistence of localized σ-type dangling bond defect sites and localized π-type paramagnetic centers associated with aromatic domains.^41^ The temperature-dependent spectra for the −0.9 V sample are shown in Figure 8c. The spectra follow similar behavior to pristine CBHPC: as the temperature increases from 10 to 100 K, the EPR signal intensity and the line width both decreases. This is also observed at other potentials (see Figure S11). Figure 8d represents the D.I. signal of the −0.9 V EPR data as a function of temperature, and results in a good fit to the Curie–Weiss equation. Other polarization voltages are plotted in Figure S12, and fitting parameters are given in Tables S8–S12. Overall, these results further confirm the coexistence of localized σ electrons centered on the carbons, associated with sp^3^-type defects and dangling bonds (Line-1, narrow line), and localized π electrons originating from graphitic-like domains (Line-2, broad line) within the CBHPC framework.^69^

Examining the EPR spectra at different potentials, it is observed that there is a clear change in Δ*H*_pp_ with the polarization potential (Figure 9). Both Line-1 and Line-2 exhibit a decrease in the Δ*H*_pp_ values within the potential range of 0 V to −0.5 V, reaching a minimum at −0.5 V. From −0.5 V to −0.9 V, the Δ*H*_pp_ values begins to increase, attaining a maximum at −0.9 V. CBHPC polarized at 0 V indicates Δ*H*_pp_ values for Line-1 and Line −2 of 6.8 and 24.1 G, respectively. Afterward it decreases to 3.7 and 14.8 G at −0.5 V for Line-1 and Line-2, respectively.

**Figure 9:**
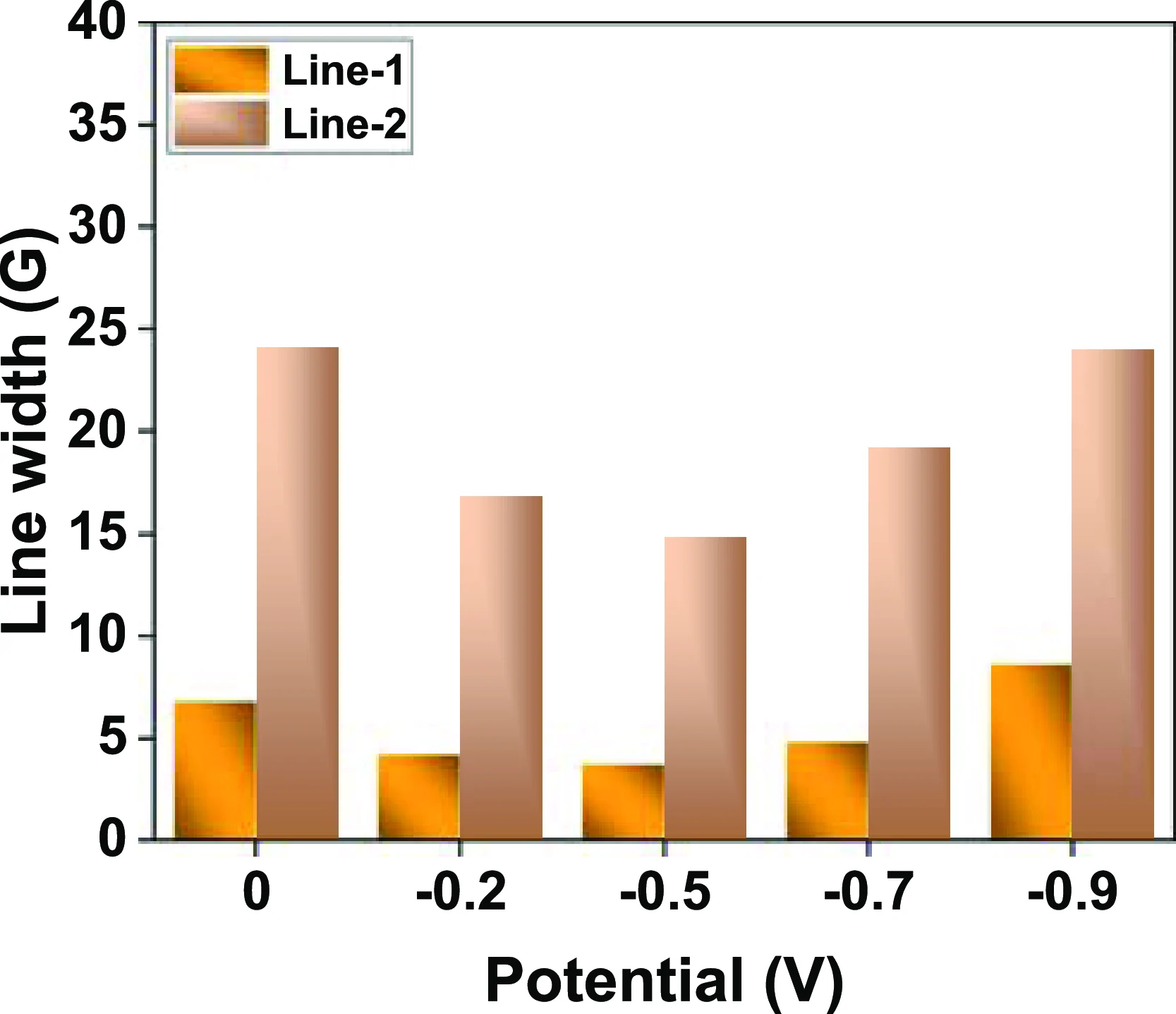
Line width of Line-1 and Line-2 as a function of the applied potentials at 10K.

In EPR, the line width is inversely proportional to spin–spin (*T*_2_) relaxation times, indicating the extent of the interaction between paramagnetic center.^70^ In this work, the decrease in Δ*H*_pp_ values for both lines with the increase of the negative applied potential (up to −0.5 V) suggests that the interaction between Na^+^ ions and carbon-centered defects is relatively weak (narrow lines often correspond to long *T*_2_ resulting in weak interactions^70^), with no significant charge transfer between the carbon centers and the Na^+^ ions. Under this condition, the Na^+^ ions likely remain in a charged (ionic) or monodispersed state. This observation suggests that within this potential range (0 V to −0.5 V), the charge storage mechanism is controlled by EDLC, driven by adsorption of Na^+^ ions on the carbon surfaces with a partial contribution from ion insertion/intercalation along with EDLC in the charge storage process. At more negative polarization potentials (−0.7 V to −0.9 V), the peak-to-peak line width trend reverses. At −0.7 V, the Δ*H*_pp_ line widths broaden to 4.8 G (Line-1) and 19.2 G (Line-2), and this broadening continues at −0.9 V, reaching 8.6 and 24.0 G for Line-1 and Line-2, respectively, at 10 K. Details of the fitting values are given in Tables S6 and S7. The larger line widths of these resonances suggested a faster *T*_2_,^71^ arising from stronger interactions between electron spins and neighboring paramagnetic centers through dipole,^72^ exchange,^73^ and chemical interactions.^74^ Thus, in this potential range, incoming Na^+^ ions are strongly interacting with carbon center defects. Na^+^ ions could transfer the charge to form transient C–Na bonds^19^ or aggregate with each other. Moreover, the broadening may arise from the development of localized paramagnetic centers in distinct local environments that differ from each other due to differences in the amount of Na^+^ nearby. Such centers may originate from Na^+^ interactions with graphene edges, intercalation between turbostratic carbon layers, and filling of ultramicropores or nanopores.^41^ The observations above suggest that, with increasing negative potential (from −0.5 V to −0.9 V), Na^+^ ions are more likely to engage in stronger interactions, such as bond formation or aggregation. In this potential range, Faradaic charge transfer processes dominate the charge storage mechanism, mediated by Na^+^ insertion/intercalation. The Δ*H*_pp_ follows a similar trend with increasing temperature (20 K–100 K) (Figure S13–21). However, the difference between the Δ*H*_pp_ values decreases at higher temperatures due to thermal motion, leading to partial averaging of the local magnetic environments.

Within the potential range from −0.5 V to −0.9 V, Na^+^ insertion/intercalation dominates the charge storage mechanism. Distinguishing between insertion and intercalation based solely on these observations is challenging, so g-value analysis is employed. Previous studies have shown that g values shift toward the free-electron g_e_ factor (*g*_e_ ≈ 2.0023) when Li^+^ intercalates between graphene layers^18,75^ In our system, the sample polarized at −0.9 V exhibits a g value of 2.0031, close to *g*_e_, whereas samples measured at 10 K under other polarization conditions display higher *g* values (in the range of 2.0045–2.0057). This shift suggests that at this potential Na^+^ ions preferentially intercalate within graphene layers rather than remaining at defect sites.

#### 3.2.4 Correlation between MAS NMR and EPR Results

Solid-state NMR revealed that at initial potentials (up to −0.2 V), the charge storage mechanism in these materials is predominantly characterized by Na^+^ adsorption at defect sites, concurrent with Na^+^ intercalation into the graphene layers. However, the detection of a significant amount of dispersed (nonaggregated) Na^+^ species suggests that ion adsorption is the dominant mechanism over intercalation. This is supported by EPR, which shows that Na^+^ ions remain largely localized, with weak interactions with carbon-centered defects and no significant charge transfer. Up to −0.5 V, a persistent, strong ^23^Na-NMR signal for monodispersed Na^+^ ions confirms that the adsorption-driven EDLC mechanism is the predominant charge-storage mechanism. This predominance is also reflected in EPR, where the continuous decrease in Δ*H*_pp_ indicates weak interactions between Na^+^ ions and carbon-centered defects. The ^23^Na NMR spectrum at −0.7 V shows the appearance of more strongly interacting Na^+^ species. In parallel, the EPR data show an increase in Δ*H*_pp_, consistent with stronger interactions between the ions and the carbon framework. These results together suggest that in this potential range, Na^+^ insertion becomes the main charge-storage mechanism, most likely via a pore-filling process. At −0.9 V, a peak appears at 7.9 ppm, consistent with Na^+^ ion intercalation between graphene layers, suggesting this mechanism is dominant at this potential. This is further supported by the continued broadening of Δ*H*_pp_ in the EPR spectra, which also exhibits g-values closer to *g*_e_. These two trends point to stronger interactions between the Na^+^ ions and the carbon defect sites. Overall, the data in Table 1 suggest a sequential evolution of the charge-storage mechanism in CHBC across different potential ranges. These changes are shown schematically in Figure 10. In the initial sloping region (0 to −0.2 V), charge storage is mainly governed by Na^+^ adsorption at the surface, along with SEI formation. At −0.5 V, adsorption-driven EDLC becomes more pronounced. As the potential decreases further toward the plateau region (up to −0.7 V), Na^+^ insertion into nanopores becomes the dominant storage process. Finally, the re-emergence of the sloping region in the voltage profile (up to −0.9 V) is associated with the intercalation of Na^+^ ions within graphene–graphene interlayer spaces.

**Figure 10:**
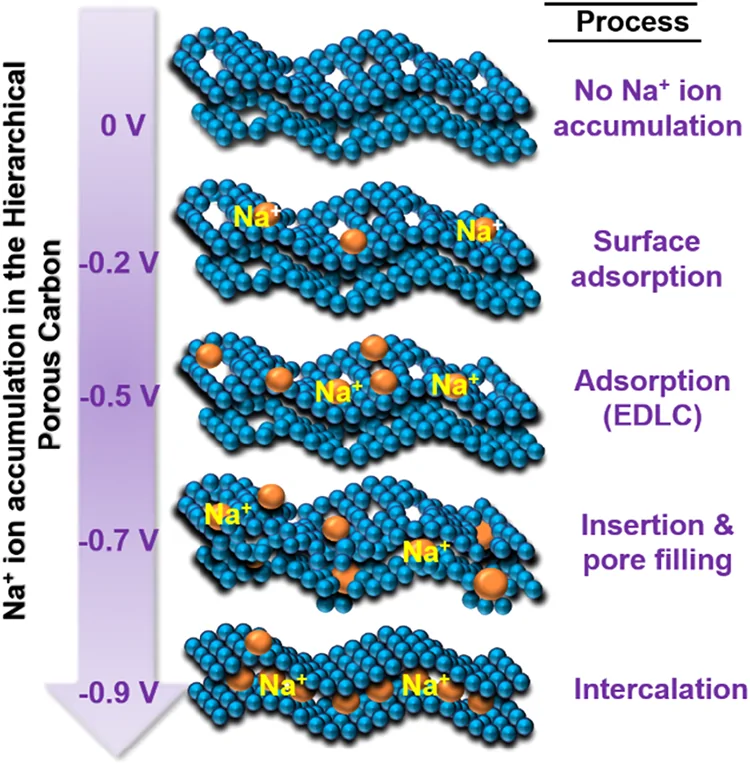
Illustration of how the sodium-storage mechanisms change with applied potential.

## 4 Conclusions

This work investigates the sodium-ion storage mechanisms in CBHPC anodes for ASIBs, with the goal of linking carbon architecture to charge-storage behavior at the molecular level. Hierarchical porous carbons are widely considered promising anodes for sodium-ion batteries, but the details of how sodium is stored in these disordered, defect-rich structures, especially in aqueous systems, are still not fully understood. In this work, CBHPC was prepared using a silica hard-templating approach, producing bowl-shaped carbon particles with a conjugated framework. Structural characterization confirms the key features of the material: XRD, Raman, BET, SEM, TEM, XPS, and ^13^C MAS NMR all point to a highly porous structure with large surface area, the presence of graphitic domains, and numerous defect sites. Combined, these characteristics make CBHPC a useful model system for the study of sodium storage mechanisms. The material delivers a reversible capacity of ∼250 mAh/g at 0.1 A/g in aqueous NaClO_4_. Cyclic voltammetry and galvanostatic charge–discharge measurements show that sodium storage involves both capacitive and diffusion-controlled processes, depending on the potential range. ^23^Na solid-state MAS NMR combined with temperature-dependent CW-EPR were used in concert to directly probe sodium storage. ^23^Na-MAS NMR distinguished sodium environments as a function of polarization potential and revealed the progressive evolution of dominant sodium-storage mechanisms. At the starting potential, Na^+^ ions adsorb onto the electrode surface through SEI formation. At moderately negative potentials, Na^+^ ions adsorb at graphene edge sites, giving rise to an EDLC-type storage mechanism. With further increase in negative potential, Na^+^ ions preferentially insert into the nanopores, and finally intercalate between graphene layers at the most negative potentials (−0.9 V). EPR provided complementary information on the electronic and defect environment of the carbon framework. Changes in the line width (Δ*H*_pp_) and *g* values provided valuable insight into the nature of Na^+^-carbon interactions, whether they are present as weakly or strongly interacting states. At the initial potentials, Δ*H*_pp_ shows a decreasing trend, indicating predominantly weak Na^+^-carbon interactions consistent with adsorption dominant EDLC mechanism. Upon further decrease in potential (down to −0.9 V), stronger Na^+^-carbon interactions are indicated, corresponding to Faradaic processes such as Na^+^ insertion and intercalation, possibly accompanied by transient charge transfer and/or aggregation effects. By correlating the electrochemical behavior with NMR and EPR signatures, this work establishes a sequential sodium storage mechanism in CBHPC: adsorption at defect-rich carbon surfaces, followed by sodium insertion into pores through pore filling, and finally intercalation between turbostratically stacked graphene layers at the most negative potentials. This approach establishes an experimental evidence-based mechanistic understanding of Na^+^ storage in the aqueous sodium-ion system, rather than relying on assumptions inferred from nonaqueous sodium-ion systems.

In this work, we also demonstrated that magnetic resonance techniques (both EPR and NMR) provide a versatile way to understand the underlying charge-storage mechanisms in carbon-based electrodes. MAS NMR was used to study the local environment of Na^+^ ions, revealing their localization within pores and their chemical interactions with the carbon framework. This approach identified distinct electrochemical processes associated with the evolution of sodium environments across different potential ranges. In addition we establish a correlation between the EPR spectral line widths of the carbon materials and charge storage processes, including changes in Na^+^-carbon interactions throughout the different potential ranges. The observed variations in EPR spectral line width provide insight into Na^+^-carbon interactions and offer a means to distinguish between capacitive and charge-transfer contributions to the charge-storage mechanism. This study also demonstrates a tool for identifying the charge storage pathways of battery materials by combining MAS NMR and EPR spectroscopy. This integrated magnetic resonance spectroscopic approach offers a model for direct and evidence-based mechanistic investigations of electrode materials. This study, therefore, provides one of the first direct magnetic-resonance-based mechanistic descriptions of sodium storage in an aqueous sodium-ion battery carbon anode. More broadly, it shows that bowl-shaped hierarchical porous carbon is not only a high-performance anode architecture but also an effective model platform for understanding how pore structure, graphitic domains, and defect chemistry govern sodium-ion storage. These findings provide mechanistic guidance for the rational design of next-generation carbon anodes for safe, low-cost, and scalable aqueous sodium-ion batteries.

## Supplementary Material



## Notes

SEM image of silica nanoparticles; core-level XPS spectra of CBHPC: (a) C 1s and (b) O 1s, ^13^C NMR spectrum of the pristine CBHPC; the *g* values, line widths and Dyssonian asymmetry ratios extracted from the EPR data collected on pristine CBHPC in the range of 10 to 100 K, and at room temperature; the fitting parameters obtained from applying the Curie–Weiss equation to the double integration (DI) of the EPR data versus temperature, from the pristine CBHPC; The dependence of the peak current (*i*_p_) from the CV curves of CBHPC at various scan rates (*v*) in the full range of 0.1–100 mV/s, and in the more specific range of 5 to 100 mV/s; Dunn's method for quantifying the charge-storage contributions; Variations in Coulombic efficiency across various current densities vs. cycle number; ^23^Na NMR of CBHPC samples charged at different potentials, without washing, overlaid with its fit; mass-normalized ^23^Na NMR of CBHPC samples charged at different potentials, after washing; Room temperature EPR spectrum of CBHPC polarized at 0, −0.2, −0.5, and −0.7 V; The *g* values, line widths and Dyssonian asymmetry ratios extracted from the EPR data collected over the range of 10 to 100 K, and at room temperature from CBHPC polarized to 0.0, −0.2, −0.5, −0.7 and −0.9 V; EPR spectrum of CBHPC polarized at 0, −0.2, −0.5, and −0.7 V at 10K; EPR spectra of CBHPC polarized at 0, −0.2, −0.5, and −0.7 V potentials, recorded at various temperatures in the range 10K −100 K; the double integration values from lines 1 and 2 plotted as a function of temperature for samples polarized to 0, −0.2, −0.5, and −0.7 V; the fitting parameters obtained from applying the Curie–Weiss equation to the DI values versus temperature data of CBHPC polarized at 0.0, −0.2, −0.5, −0.7 and −0.9 V; the line widths of lines 1 and 2 from the EPR fittings, plotted as a function of the applied potentials at 20, 30, 40, 50, 60, 70, 80, 90, and 100 K (PDF)
