# Supplementary material for: Elucidating Sodium-Ion Storage Mechanisms in Bowl-Shaped Hierarchical Porous Carbon Anodes: A Combined Solid-State NMR and EPR Approach
Source: J Am Chem Soc. 2026 Sep 8;148(37):39565–79. doi: 10.1021/jacs.6c08138 (PMC13614179; doi:10.1021/jacs.6c08138)
Supplement: Supplementary file 1 [file ja6c08138_si_001.pdf]

**Supporting Information**

# Elucidating Sodium-Ion Storage Mechanisms in Bowl-Shaped Hierarchical Porous Carbon Anodes: A Combined Solid-State NMR and EPR Approach

*Ananya Pal[1], Nicole Leifer[2], Daphna Shimon[1*]*

1 Institute of Chemistry, The Hebrew University of Jerusalem, Jerusalem 9190401, Israel

2 Department of Chemistry, Institute of Nanotechnology and Advanced Materials, Bar-Ilan University, INIES - Israel National Institute for Energy Storage, supported by Israel Ministry of Energy and Infrastructures., Ramat Gan, 5290002 Israel

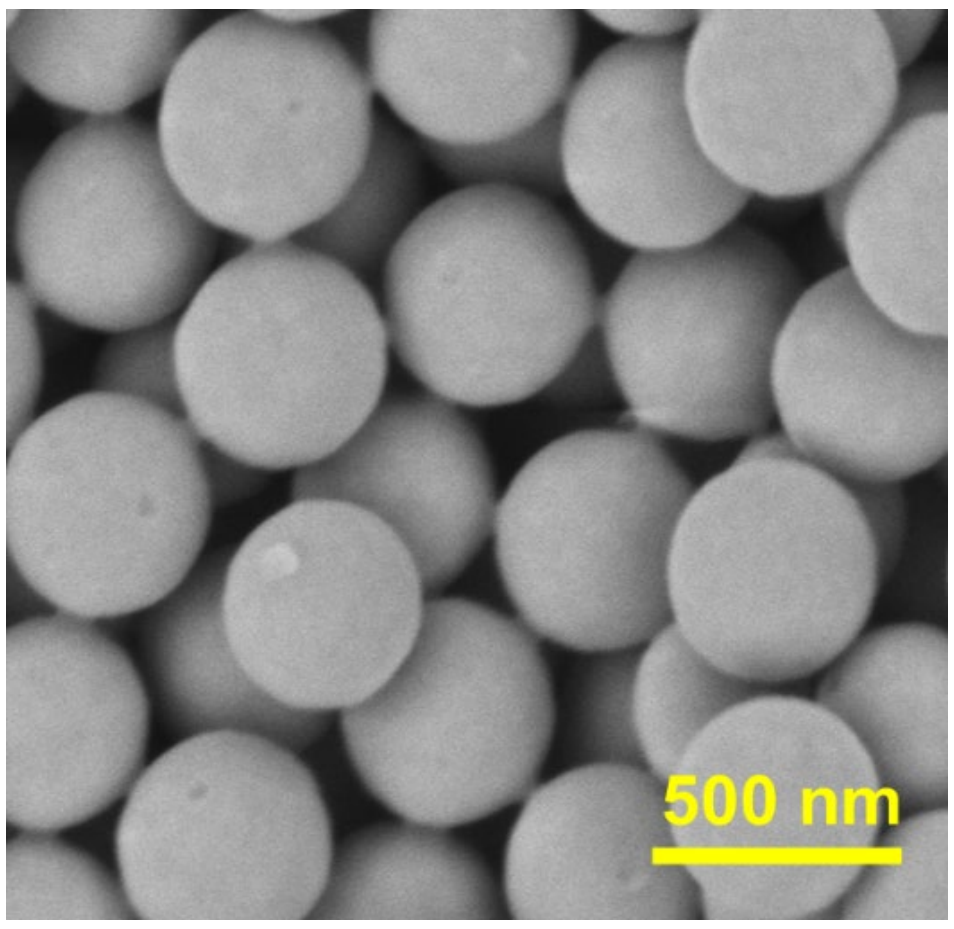


**Supporting Information Figure S1**.SEM image of S-$SiO_2$ nanoparticles before functionalization. These particles serve as a template for the assembly of the bowl-shaped carbon material.

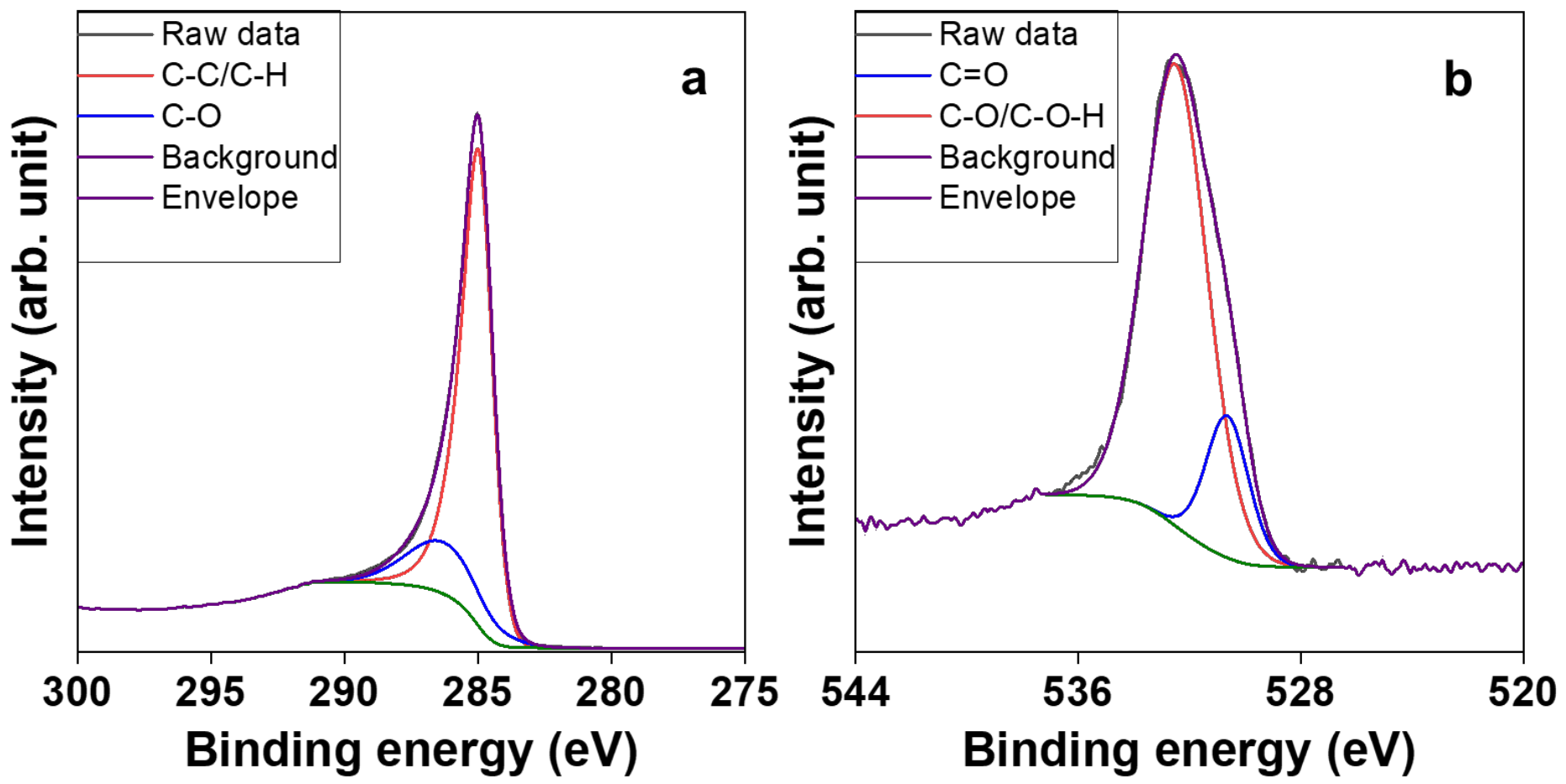


**Supporting Information Figure S2.** Core-level XPS spectra of CBHPC: (a) C 1s and (b) O 1s. The C 1s spectra indicate peaks at approximately 285.0 eV, consistent with the C-C and C-H bonds in a carbon framework, along with an additional peak at 286.3 eV, indicative of C-O bonds. The O 1s spectrum indicates the presence of C=O, C-O/C-O-H functionality in the CBHPC framework with peaks at 530.6 eV and 532.6 eV, respectively.

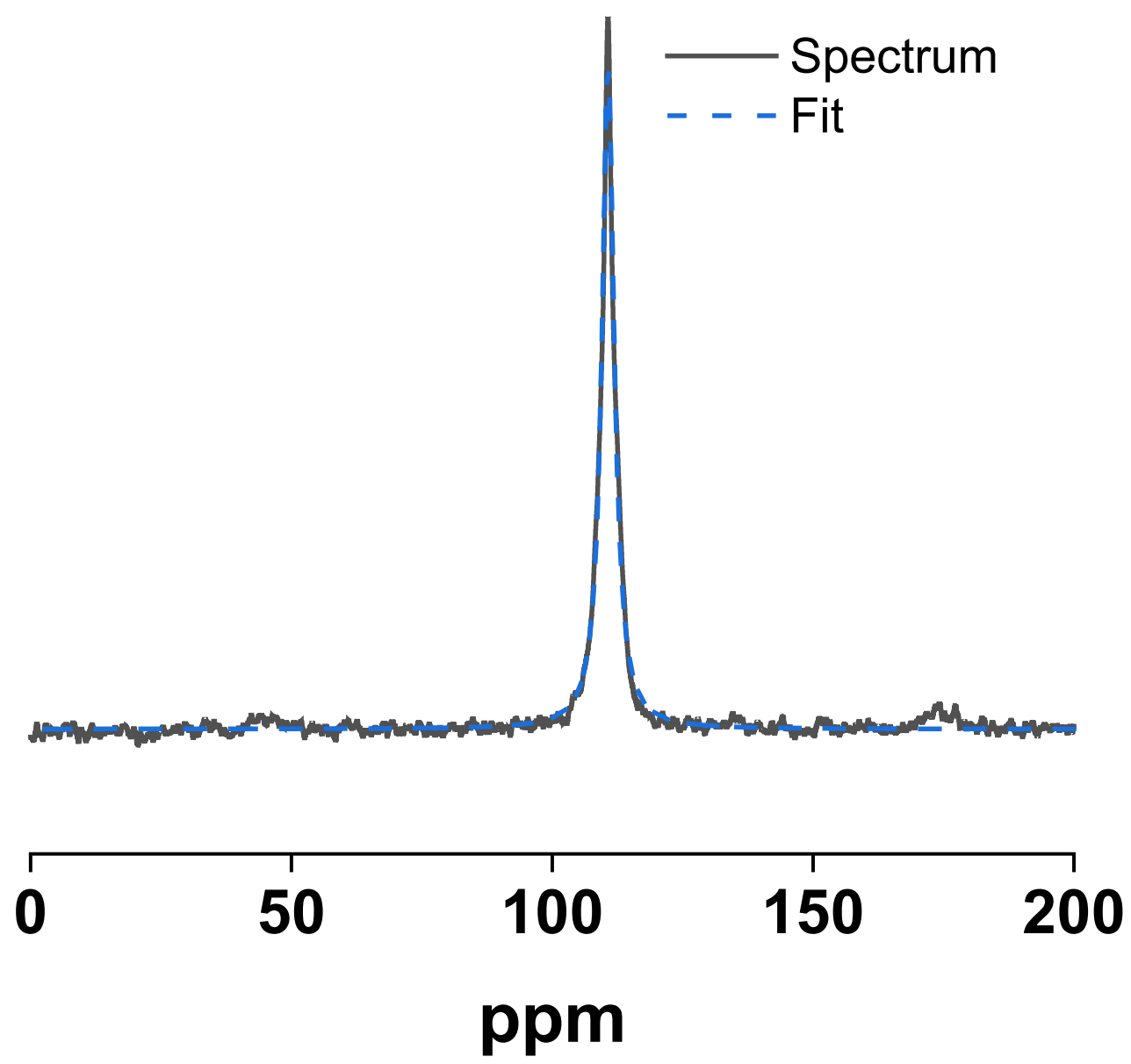


**Supporting Information Figure S3.** The $^{13}C$ NMR spectrum of the pristine CBHPC (black), overlaid with its fit (dashed blue). The appearance of a single peak at 111.7 ppm is indicative of $sp^2$-hybridized carbons within a carbon framework.

**Supporting Information Table S1:** The g values, line widths, and Dyssonian asymmetry ratios extracted from the EPR data collected on pristine CBHPC in the range of 10 K to 100 K, and at room temperature. The changes in line widths are consistent with motional narrowing due to thermally activated spin motion averaging out local field inhomogeneities, and the low asymmetry values indicate that a significant contribution to the signal originates from localized paramagnetic centers, consistent with the presence of partially delocalized electrons in CBHPC.

| **Temperature (K)** | **g values of Line-1** | **g values of Line-2** | **$\Delta H_{pp}$ value of Line -1 (G)** | **$\Delta H_{pp}$ value of Line -2 (G)** | **Asymmetry Calculation*** |
|---|---|---|---|---|---|
| 10 | 2.0037 | 2.0037 | 6.0 | 20.9 | 1.1287 |
| 20 | 2.0034 | 2.0030 | 4.9 | 19.6 | 1.0623 |
| 30 | 2.0030 | 2.0030 | 4.8 | 18.0 | 0.9801 |
| 40 | 2.003 | 2.0037 | 4.6 | 19.2 | 0.9800 |
| 50 | 2.0032 | 2.0037 | 4.5 | 16.0 | 0.9413 |
| 60 | 2.0027 | 2.0027 | 4.0 | 14.0 | 0.9225 |
| 70 | 2.0027 | 2.0029 | 3.5 | 13.6 | 0.9605 |
| 80 | 2.0028 | 2.0032 | 3.3 | 12.0 | 0.9605 |
| 90 | 2.0028 | 2.0032 | 3.2 | 11.6 | 0.9414 |
| 100 | 2.0028 | 2.0030 | 2.8 | 11.2 | 1.0204 |
| Room Temp. | 2.0055 | 2.0040 | 6.1 | 35.2 | 0.8339 |

*A/B ratio corresponding to the low-field and high-field peak amplitudes.

**Supporting Information Table S2**: The fitting parameters obtained from applying the Curie-Weiss equation to the double integration (DI) of the EPR data versus temperature, from the pristine CBHPC.

| | Line-1 | Line-2 |
|---|---|---|
| $\chi_{Pauli}$ (arb. unit) | -21.4±10.6 | -8.4±5.4 |
| C (K) | 2729.5±768.7 | 2111.0±240.5 |
| $T_0$(K) | -4.3±3.6 | 4.5±0.6 |
| $R^2$ | 0.9982 | 0.9991 |

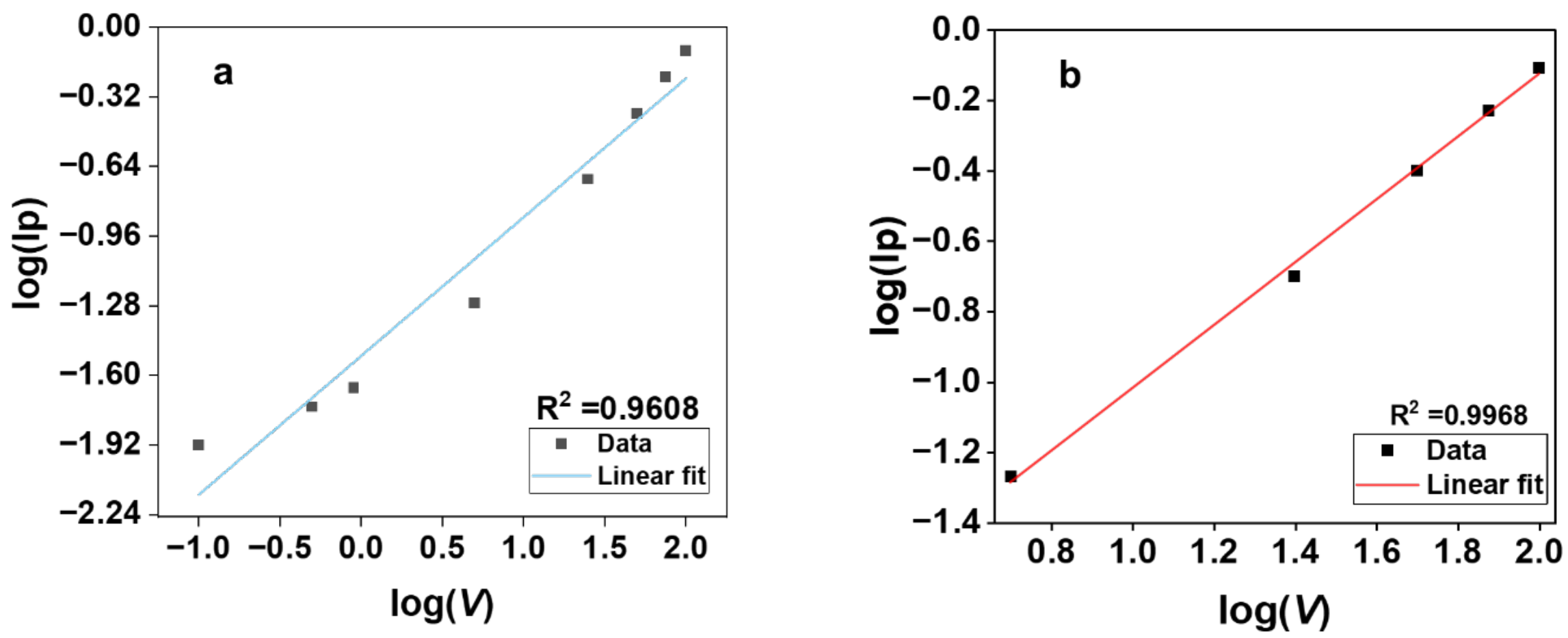


**Supporting Information Figure S4.** The dependence of the peak current ($i_p$) from the CV curves of CBHPC at various scan rates ($v$) in the full range of (a) 0.1-100 mV /s, and in the more specific range of (b) 5 to 100 mV/s. The experimental data (black) is overlaid with a linear fit (blue/red). At lower potentials (first half of graph a), the data indicate that the sodium storage process is predominantly diffusion-controlled. At higher scan rates (graph b), the data suggest both chemical adsorption and sodium insertion.

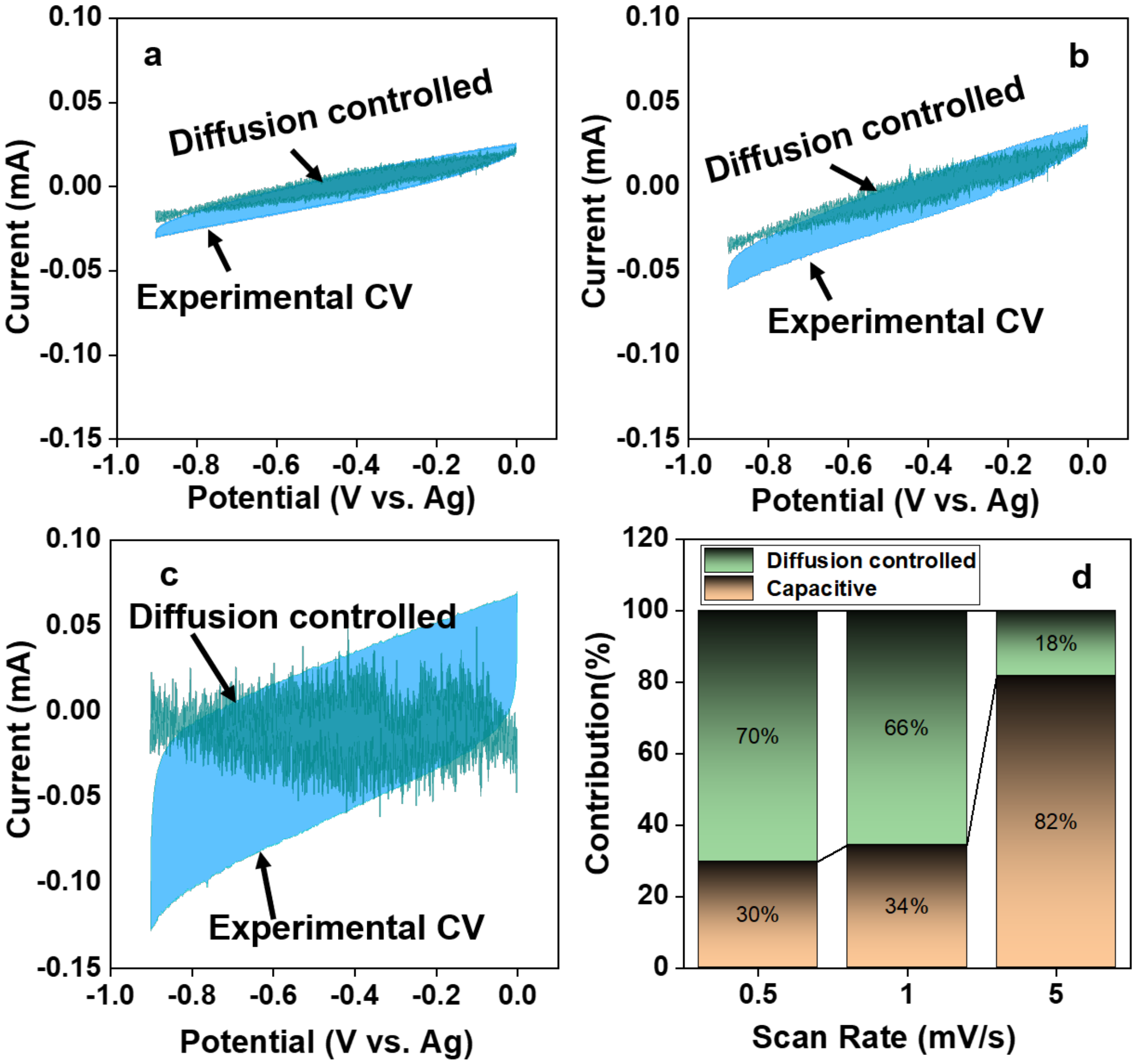


**Supporting Information Figure S5.** Dunn's method for quantifying the charge-storage contributions using the equation $i(V)= k_1v + k_2v^{0.5}$, where $k_1v$ *and* $k_2v^{0.5}$ represent the capacitive contributions and diffusion-controlled contribution, respectively. a) 0.5 mV/s, b) 1 mV/s, c) 5 mV/s, and d) percentage of capacitive and diffusive contributions at different scan rates. These data reveal that at low scan rates diffusion-controlled processes predominate, and at high scan rates capacitive-controlled processes predominate.

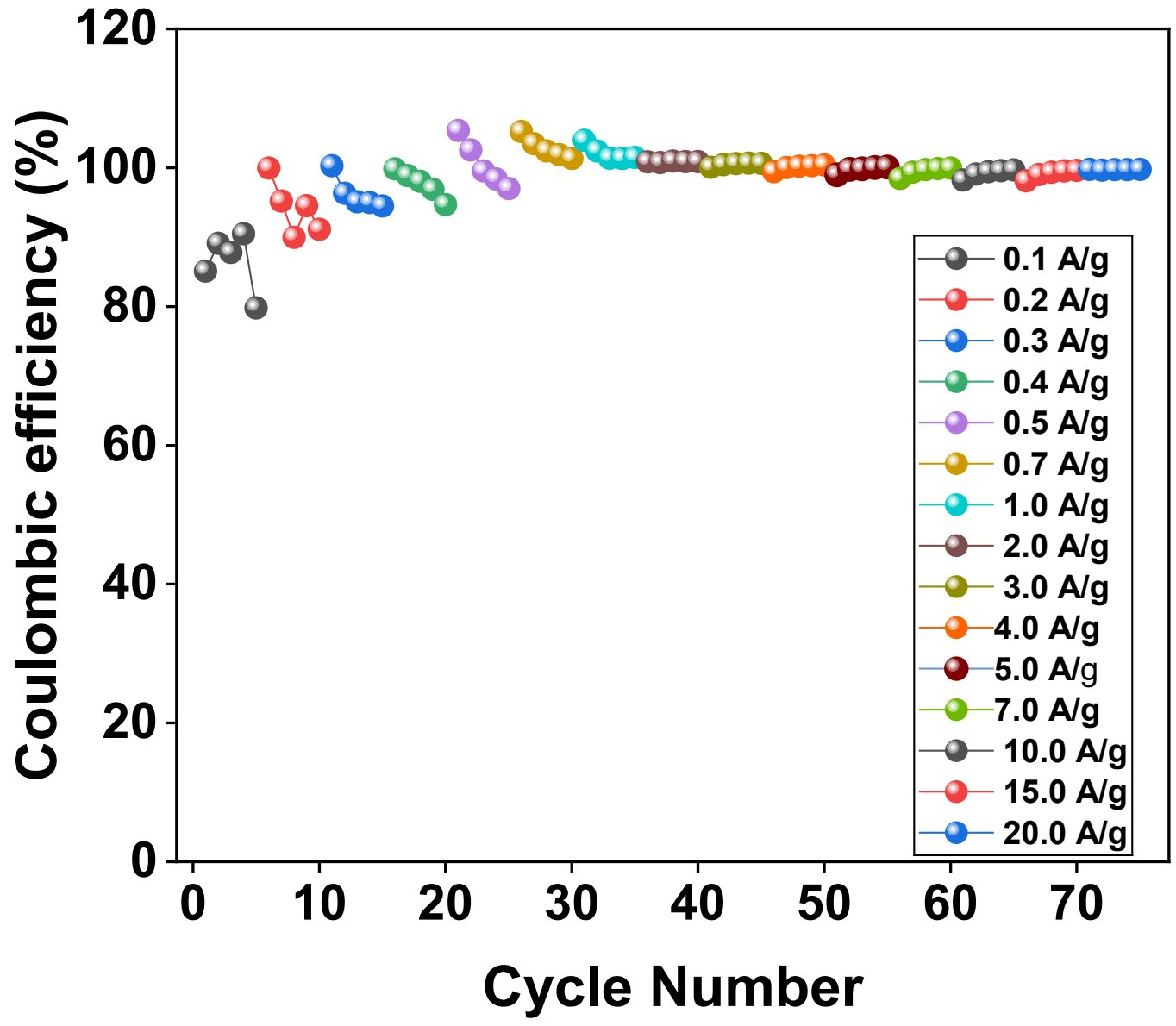


**Supporting Information Figure S6.** Variations in Coulombic efficiency (CE) across various current densities vs. cycle number. The CE progressively increases with increasing current density and reaches ~100% at moderate to high current densities.

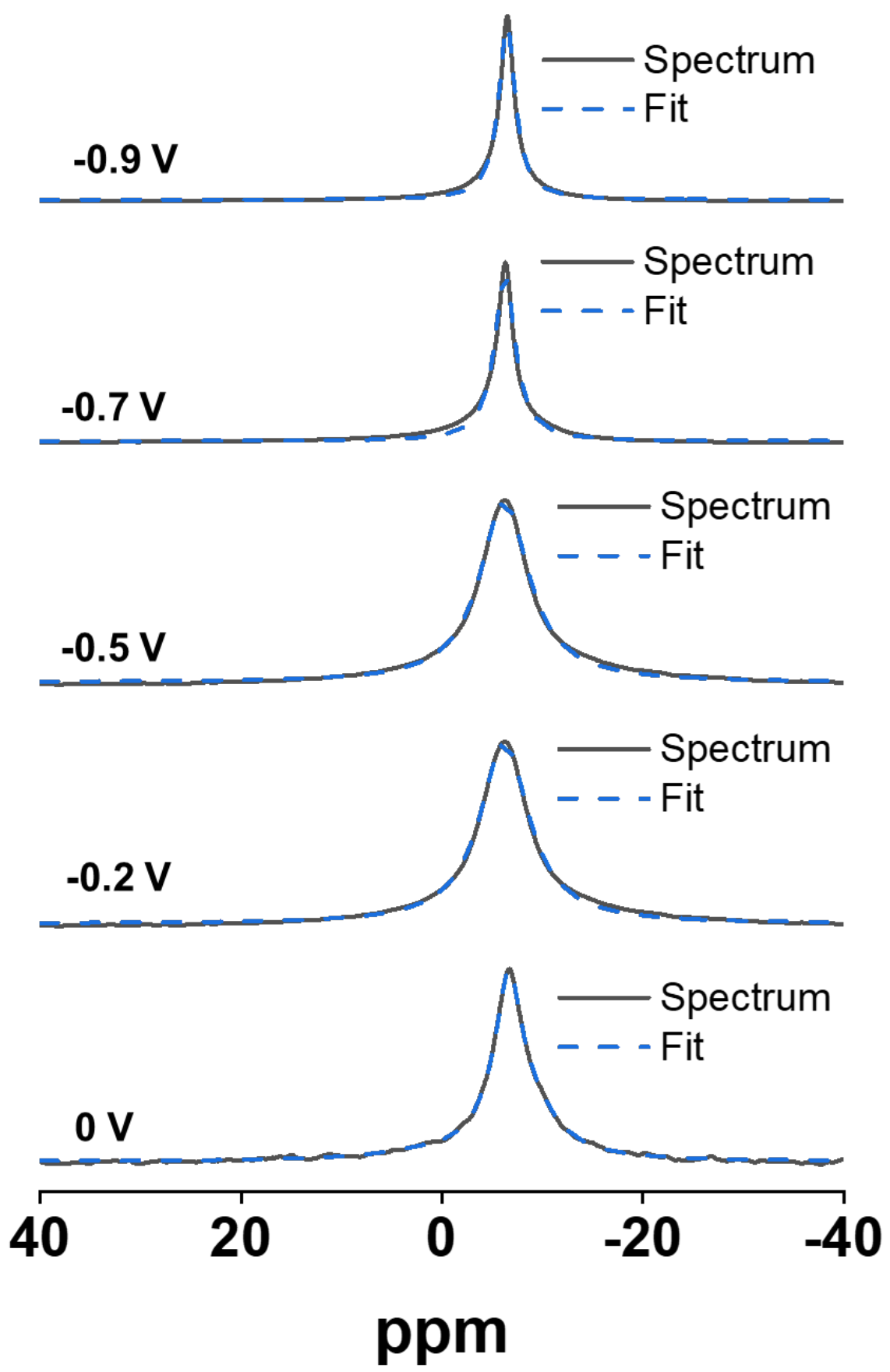


**Supporting Information Figure S7.** The $^{23}$Na NMR of CBHPC samples charged at different potentials, without washing (black), overlaid with their fit (dashed blue). The unwashed samples all exhibited a strong resonance peak at -6.5 ppm, corresponding to residual electrolyte, which obscures the other peaks in the spectrum.

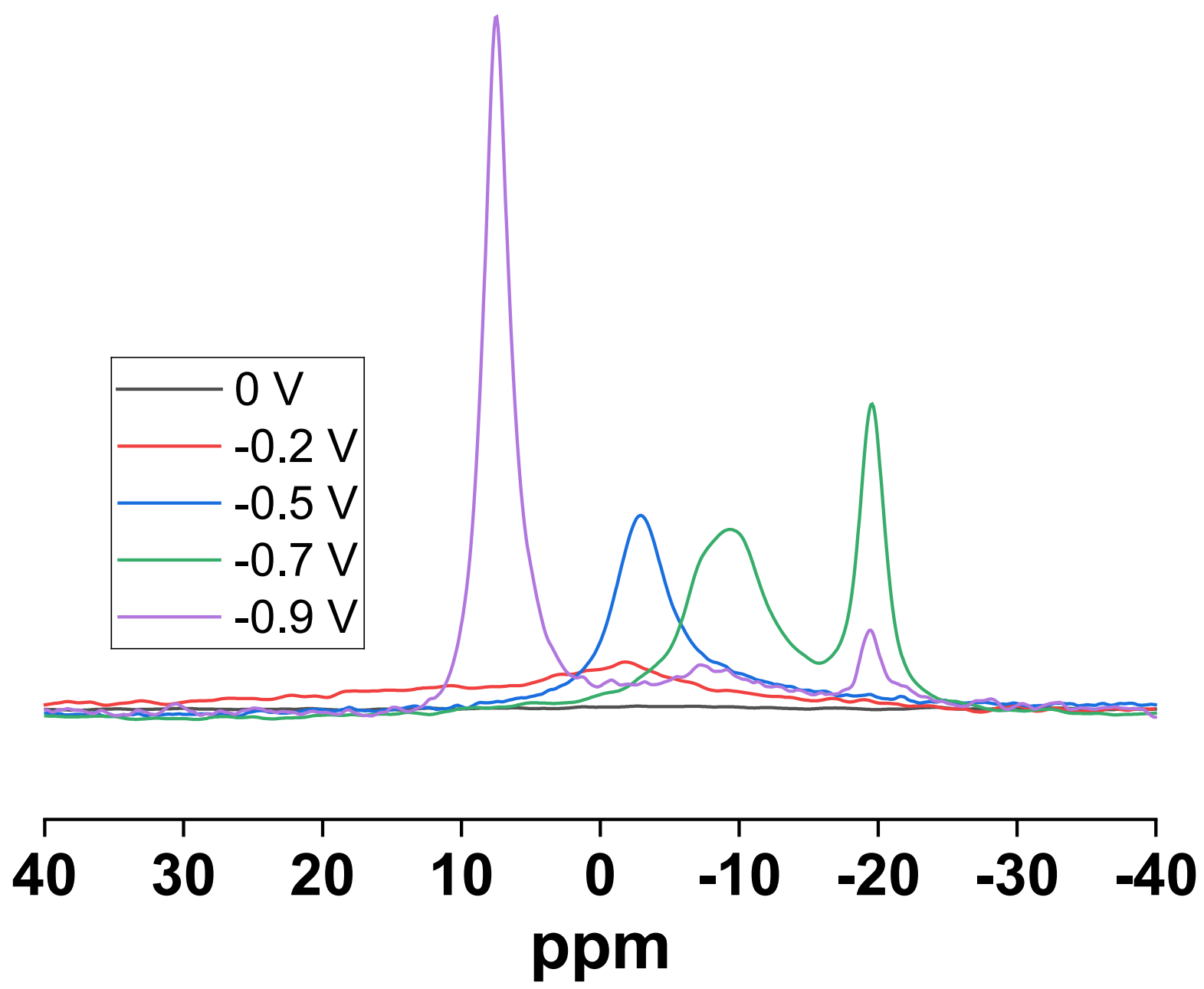


**Supporting Information Figure S8.** The mass-normalized $^{23}Na$ NMR of CBHPC samples charged at different potentials, after washing. This shows a concomitant increase in peak intensity with an increase in the applied negative polarization potential. In other words, $Na^+$ ion accumulation on the carbon increases as the potential is lowered to more negative values.

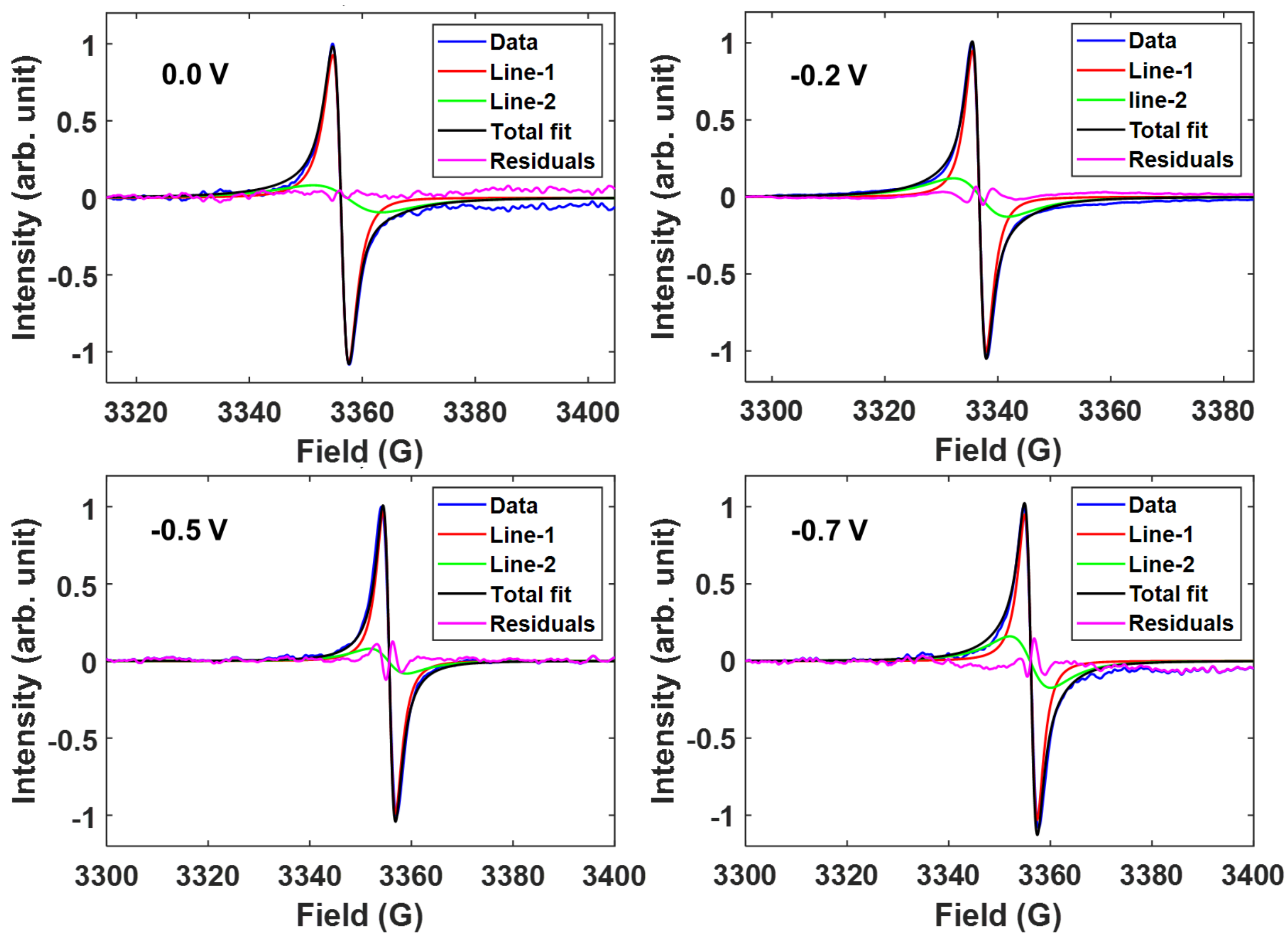


**Supporting Information Figure S9.** Room temperature EPR spectrum of CBHPC polarized at 0 V, -0.2 V, -0.5 V, and -0.7 V (blue). All spectra are fitted using a two-component (two-line) model (red, green). The total fit (black) and the residuals (magenta) are also overlaid. All the figures show a single peak fitted with two lines. Line-1 exhibits a g-value in the range of 2.0056 -2.0057 with comparable $\Delta H_{pp}$ values across all polarization states. This line is consistent with localized σ-type dangling bonds/defects. Line-2, at RT, shows g-values that vary in the range of 2.0054 -2.006 with corresponding $\Delta H_{pp}$ values varying from 7.2 to 12.1 G for all polarization states. This broader component is likely attributed to localized π electrons originating from aromatic domains.

**Supporting Information Table S3**: The g values, line widths, and Dyssonian asymmetry ratios extracted from the EPR data collected from CBHPC polarized to 0.0 V, collected over the range of 10 K to 100 K, and at room temperature. The trends and implications are similar to those of the pristine CBHPC material.

| **Temperature (K)** | **g values of Line-1** | **g values of Line-2** | **$\Delta H_{pp}$ value of Line -1 (G)** | **$\Delta H_{pp}$ value of Line -2 (G)** | **Asymmetry Calculation*** |
|---|---|---|---|---|---|
| 10 | 2.0046 | 2.0045 | 6.8 | 24.1 | 1.1061 |
| 20 | 2.0049 | 2.0050 | 5.8 | 18.9 | 1.1061 |
| 30 | 2.0052 | 2.0050 | 5.1 | 16.8 | 1.0624 |
| 40 | 2.0053 | 2.0049 | 4.5 | 15.2 | 1.0840 |
| 50 | 2.0054 | 2.0050 | 3.8 | 11.6 | 0.9800 |
| 60 | 2.0055 | 2.005 | 3.4 | 11.2 | 0.9413 |
| 70 | 2.0055 | 2.0056 | 3.1 | 9.2 | 0.9605 |
| 80 | 2.0057 | 2.0055 | 2.5 | 9.6 | 1.0838 |
| 90 | 2.0058 | 2.005 | 2.4 | 8.1 | 0.9414 |
| 100 | 2.0058 | 2.0058 | 2.4 | 7.4 | 0.9606 |
| Room Temp. | 2.0056 | 2.006 | 2.8 | 12.1 | 0.8664 |

*A/B ratio corresponding to the low-field and high-field peak amplitudes.

**Supporting Information Table S4:** The g values, line widths, and Dyssonian asymmetry ratios extracted from the EPR data collected from CBHPC polarized to -0.2 V, collected over the range of 10 K to 100 K, and at room temperature. The trends and implications are similar to those of the pristine CBHPC material.

| **Temperature (K)** | **g values of Line-1** | **g values of Line-2** | **$\Delta H_{pp}$ value of Line -1 (G)** | **$\Delta H_{pp}$ value of Line -2 (G)** | **Asymmetry Calculation*** |
|---|---|---|---|---|---|
| 10 | 2.0057 | 2.0057 | 4.2 | 16.8 | 0.9800 |
| 20 | 2.0057 | 2.0057 | 3.5 | 12.4 | 0.9605 |
| 30 | 2.0057 | 2.0057 | 3.2 | 12.0 | 1.0410 |
| 40 | 2.0057 | 2.0057 | 3.0 | 11.2 | 1.0204 |
| 50 | 2.0057 | 2.0057 | 2.9 | 11.2 | 1.0203 |
| 60 | 2.0057 | 2.0057 | 3.6 | 10.0 | 0.9041 |
| 70 | 2.0057 | 2.0057 | 3.8 | 12.8 | 0.7234 |
| 80 | 2.0057 | 2.0055 | 2.7 | 8.0 | 0.8862 |
| 90 | 2.0057 | 2.0057 | 2.6 | 9.6 | 0.9415 |
| 100 | 2.0057 | 2.0057 | 2.5 | 7.6 | 0.98.1 |
| Room Temp. | 2.0056 | 2.0057 | 2.4 | 9.6 | 0.9821 |

*A/B ratio corresponding to the low-field and high-field peak amplitudes.

**Supporting Information Table S5:** The g values, line widths, and Dyssonian asymmetry ratios extracted from the EPR data collected from CBHPC polarized to -0.5 V, collected over the range of 10 K to 100 K, and at room temperature. The trends exhibited at this voltage also confirm the coexistence of localized σ electrons associated with defects and dangling bonds, and localized π electrons originating from graphitic-like domains within the CBHPC framework.

| **Temperature (K)** | **g values of Line-1** | **g values of Line-2** | **$\Delta H_{pp}$ value of Line -1 (G)** | **$\Delta H_{pp}$ value of Line -2 (G)** | **Asymmetry Calculation*** |
|---|---|---|---|---|---|
| 10 | 2.0057 | 2.0054 | 3.7 | 14.8 | 0.9800 |
| 20 | 2.0057 | 2.0055 | 3.4 | 12.8 | 0.9413 |
| 30 | 2.0057 | 2.0055 | 3.1 | 11.8 | 0.9612 |
| 40 | 2.0057 | 2.0052 | 2.8 | 10.0 | 0.9414 |
| 50 | 2.0050 | 2.0050 | 3.0 | 11.6 | 0.9041 |
| 60 | 2.0057 | 2.0057 | 2.8 | 8.4 | 0.9414 |
| 70 | 2.0053 | 2.0053 | 3.1 | 10.0 | 0.9042 |
| 80 | 2.0052 | 2.0053 | 2.7 | 8.0 | 0.9227 |
| 90 | 2.0055 | 2.0053 | 2.6 | 8.0 | 0.9800 |
| 100 | 2.0053 | 2.0053 | 2.5 | 8.4 | 0.9604 |
| Room Temp. | 2.0057 | 2.0057 | 2.4 | 7.2 | 0.9802 |

*A/B ratio corresponding to the low-field and high-field peak amplitudes.

**Supporting Information Table S6:** The g values, line widths, and Dyssonian asymmetry ratios extracted from the EPR data collected from CBHPC polarized to -0.7 V, collected over the range of 10 K to 100 K, and at room temperature. The trends exhibited at this voltage also confirm the coexistence of localized σ electrons associated with defects and dangling bonds, and localized π electrons originating from graphitic-like domains within the CBHPC framework.

| Temperature (K) | g values of Line-1 | g values of Line-2 | $\Delta H_{pp}$ value of Line -1 (G) | $\Delta H_{pp}$ value of Line -2 (G) | Asymmetry Calculation* |
|---|---|---|---|---|---|
| 10 | 2.0055 | 2.0057 | 4.8 | 19.2 | 0.8861 |
| 20 | 2.0056 | 2.0057 | 4.3 | 21.2 | 0.8683 |
| 30 | 2.00556 | 2.004 | 4.3 | 12.1 | 0.9041 |
| 40 | 2.0054 | 2.0048 | 4.3 | 14.9 | 0.7539 |
| 50 | 2.0050 | 2.0050 | 3.8 | 13.6 | 0.9041 |
| 60 | 2.0052 | 2.0049 | 3.7 | 12.4 | 0.9041 |
| 70 | 2.0052 | 2.0051 | 3.5 | 10.0 | 0.9042 |
| 80 | 2.0052 | 2.0054 | 3.4 | 11.2 | 0.7694 |
| 90 | 2.0055 | 2.0053 | 3.1 | 10.8 | 0.9318 |
| 100 | 2.0053 | 2.0054 | 2.9 | 10.4 | 0.9414 |
| Room Temp. | 2.0057 | 2.0057 | 2.4 | 8.0 | 0.8419 |

*A/B ratio corresponding to the low-field and high-field peak amplitudes.

**Supporting Information Table S7**: The g values, line widths, and Dyssonian asymmetry ratios extracted from the EPR data collected from CBHPC polarized to -0.9 V, collected over the range of 10 K to 100 K, and at room temperature. The trends exhibited at this voltage also confirm the coexistence of localized σ electrons associated with defects and dangling bonds, and localized π electrons originating from graphitic-like domains within the CBHPC framework.

| **Temperature (K)** | **g values of Line-1** | **g values of Line-2** | **$\Delta H_{pp}$ value of Line -1 (G)** | **$\Delta H_{pp}$ value of Line -2 (G)** | **Asymmetry Calculation*** |
|---|---|---|---|---|---|
| 10 | 2.0030 | 2.0031 | 8.6 | 24.0 | 0.9041 |
| 20 | 2.0031 | 2.0026 | 5.9 | 21.6 | 0.9801 |
| 30 | 2.0031 | 2.0027 | 5.0 | 19.2 | 0.9800 |
| 40 | 2.0030 | 2.0027 | 4.7 | 18.8 | 0.9413 |
| 50 | 2.0027 | 2.0027 | 4.2 | 16.4 | 0.9413 |
| 60 | 2.0027 | 2.0027 | 3.8 | 16.0 | 0.9800 |
| 70 | 2.0028 | 2.0026 | 3.6 | 15.2 | 0.9801 |
| 80 | 2.0027 | 2.0027 | 3.6 | 14.8 | 1.083 |
| 90 | 2.0030 | 2.0027 | 3.4 | 15.6 | 1.0203 |
| 100 | 2.0028 | 2.0027 | 3.1 | 14.0 | 1.062 |
| Room Temp. | 2.0056 | 2.0054 | 2.4 | 7.2 | 0.9412 |

*A/B ratio corresponding to the low-field and high-field peak amplitudes.

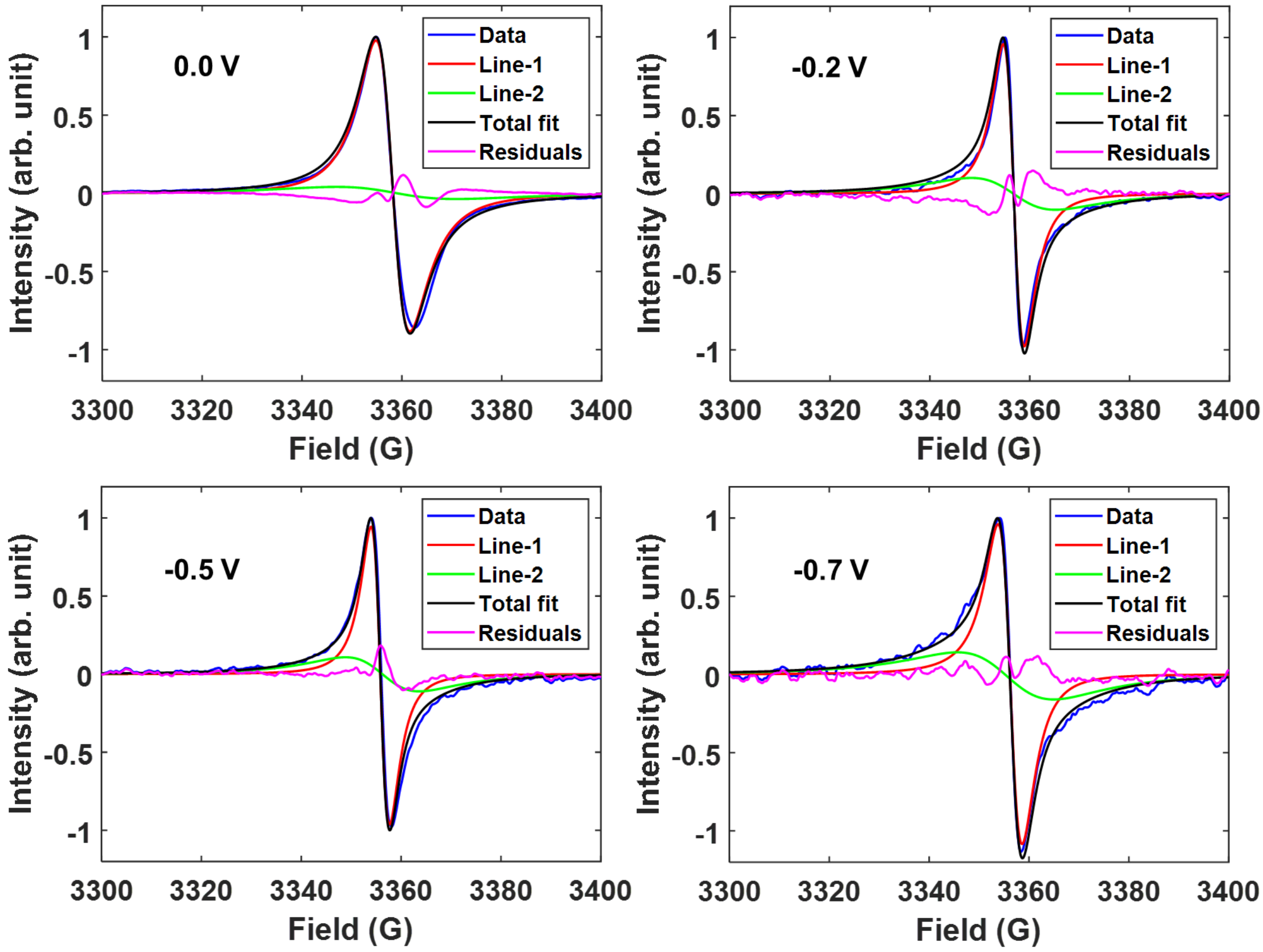


**Supporting Information Figure S10.** EPR spectrum of CBHPC polarized at 0 V, -0.2 V, -0.5 V, and -0.7 V (blue) at 10K. All spectra are fitted using a two-component (two-line) model (red, green) The total fit (black) and the residuals (magenta) are also overlaid. At each potential, the main peak could be fitted with two lines with varying line widths. The g-values for line-1 lie in the range of 2.0031-2.0057, while those for line 2 vary in the range of 2.0026-2.0057.

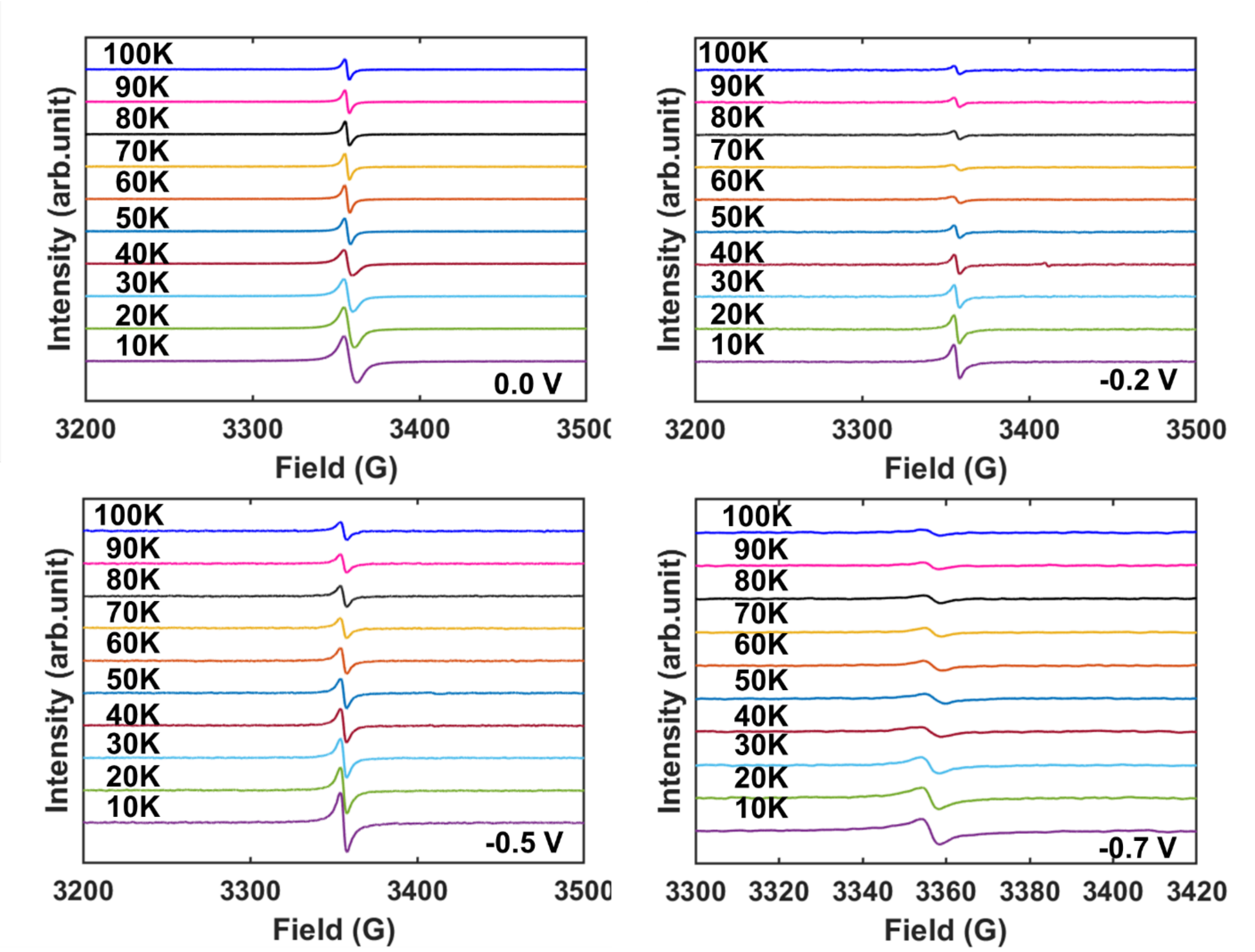


**Supporting Information Figure S11.** EPR spectra of CBHPC polarized at 0 V, -0.2 V, -0.5 V, and -0.7 V potentials, recorded at various temperatures in the range 10K -100K. These figures indicate a decrease in peak intensity and line width as the temperature is increased from 10 to 100 K. This decrease is attributed to thermal motion, which reduces the net magnetic susceptibility and consequently decreases the EPR signal intensity.

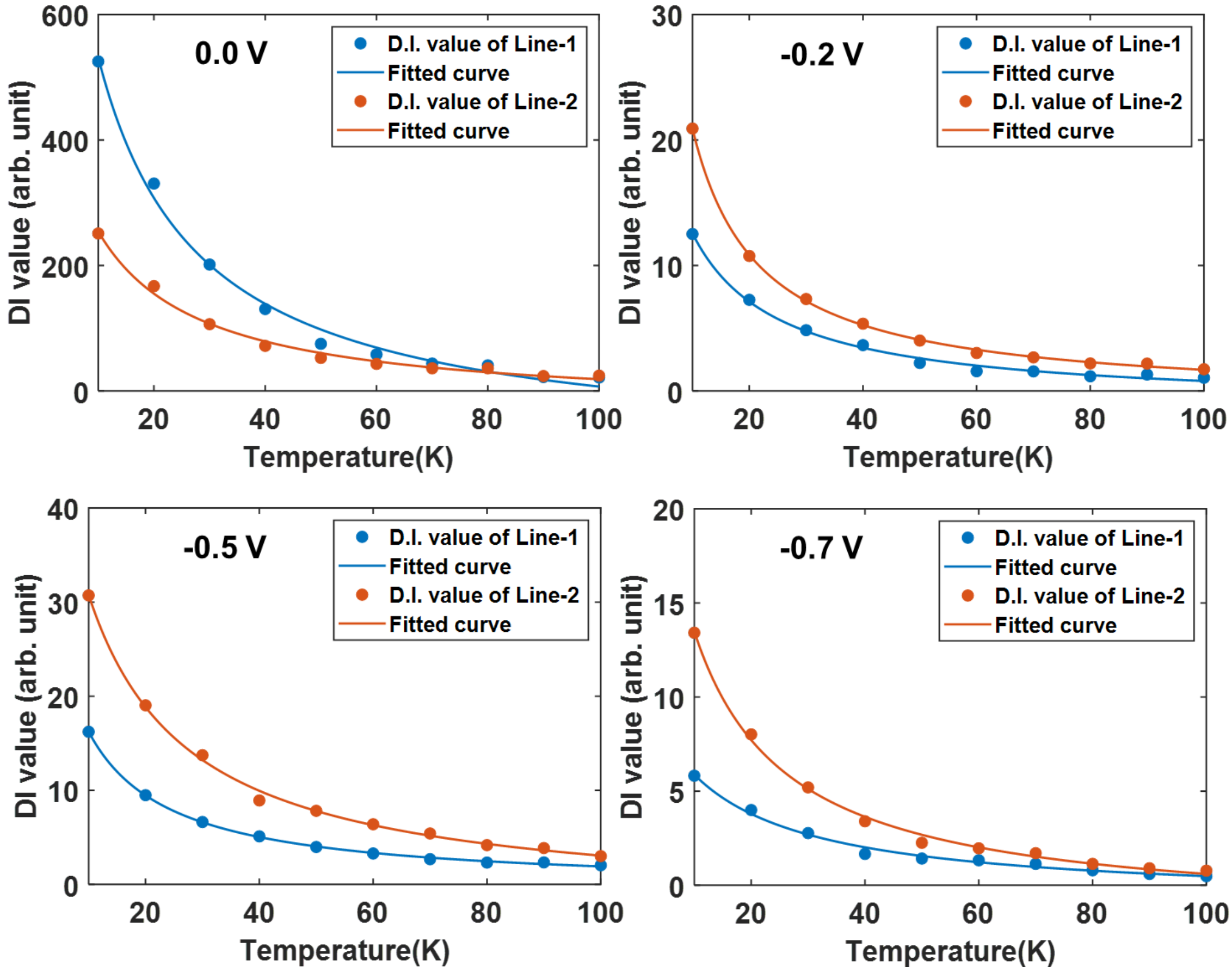


**Supporting Information Figure S12.** The DI values from line 1 (blue) and line 2 (red) are plotted as a function of temperature for samples polarized to 0 V, -0.2 V, -0.5 V, and -0.7 V. The experimental data are overlaid with a fit using the Curie-Weiss equation (solid lines). In all polarization states, both lines-1 and 2 exhibit Curie-Weiss magnetism (i.e., a gradual decrease in the magnetization upon an increase in the temperature).

**Supporting Information Table S8:** The fitting parameters obtained from applying the Curie-Weiss equation to the DI values versus temperature data of CBHPC polarized at 0.0 V

| | Line-1 | Line-2 |
|---|---|---|
| $\chi_{Pauli}$ (arb. unit) | 98.8±47.2 | -30.3±22.6 |
| C (K) | 11447± 3995 | 5325.6±1926.6 |
| $T_0$(K) | -8.15±5.4 | -8.7±5.8 |
| $R^2$ | 0.9934 | 0.9928 |

**Supporting Information Table S9:** The fitting parameters obtained from applying the Curie-Weiss equation to the DI values versus temperature data of CBHPC polarized at -0.2 V

| | Line-1 | Line-2 |
|---|---|---|
| $\chi_{Pauli}$ (arb. unit) | -1.2±0.8 | -0.8±0.7 |
| C (K) | 210.4± 59.6 | 250.7±24.1 |
| $T_0$(K) | -5.3±3.8 | -1.6±0.9 |
| $R^2$ | 0.9952 | 0.9994 |

**Supporting Information Table S10**: The fitting parameters obtained from applying the Curie-Weiss equation to the DI values versus temperature data of CBHPC polarized at -0.5 V

| | Line-1 | Line-2 |
|---|---|---|
| $\chi_{Pauli}$ (arb. unit) | -0.4±0.3 | -2.4±1.4 |
| C (K) | 242.4±24 | 592.1±116.6 |
| $T_0$(K) | -4.6±1.3 | -7.8±0.3 |
| $R^2$ | 0.9993 | 0.9978 |

**Supporting Information Table S11:** The fitting parameters obtained from applying the Curie-Weiss equation to the DI values versus temperature data of CBHPC polarized at -0.7 V

| | Line-1 | Line-2 |
|---|---|---|
| $\chi_{Pauli}$ (arb. unit) | -1.0±0.25 | -1.9±0.9 |
| C (K) | 161.6±82.1 | 264.7±72.4 |
| $T_0$(K) | -13.5±10.0 | -7.1±4.1 |
| $R^2$ | 0.9989 | 0.9972 |

**Supporting Information Table S12:** The fitting parameters obtained from applying the Curie-Weiss equation to the DI values versus temperature data of CBHPC polarized at -0.9 V

| | Line-1 | Line-2 |
|---|---|---|
| $\chi_{Pauli}$ (arb. unit) | -16.6±10.9 | -39.4±21.1 |
| C (K) | 6309.1±583.0 | 12931.0±1593.5 |
| $T_0$(K) | 1.7±0.7 | 5.5±1.7 |
| $R^2$ | 0.9995 | 0.9991 |

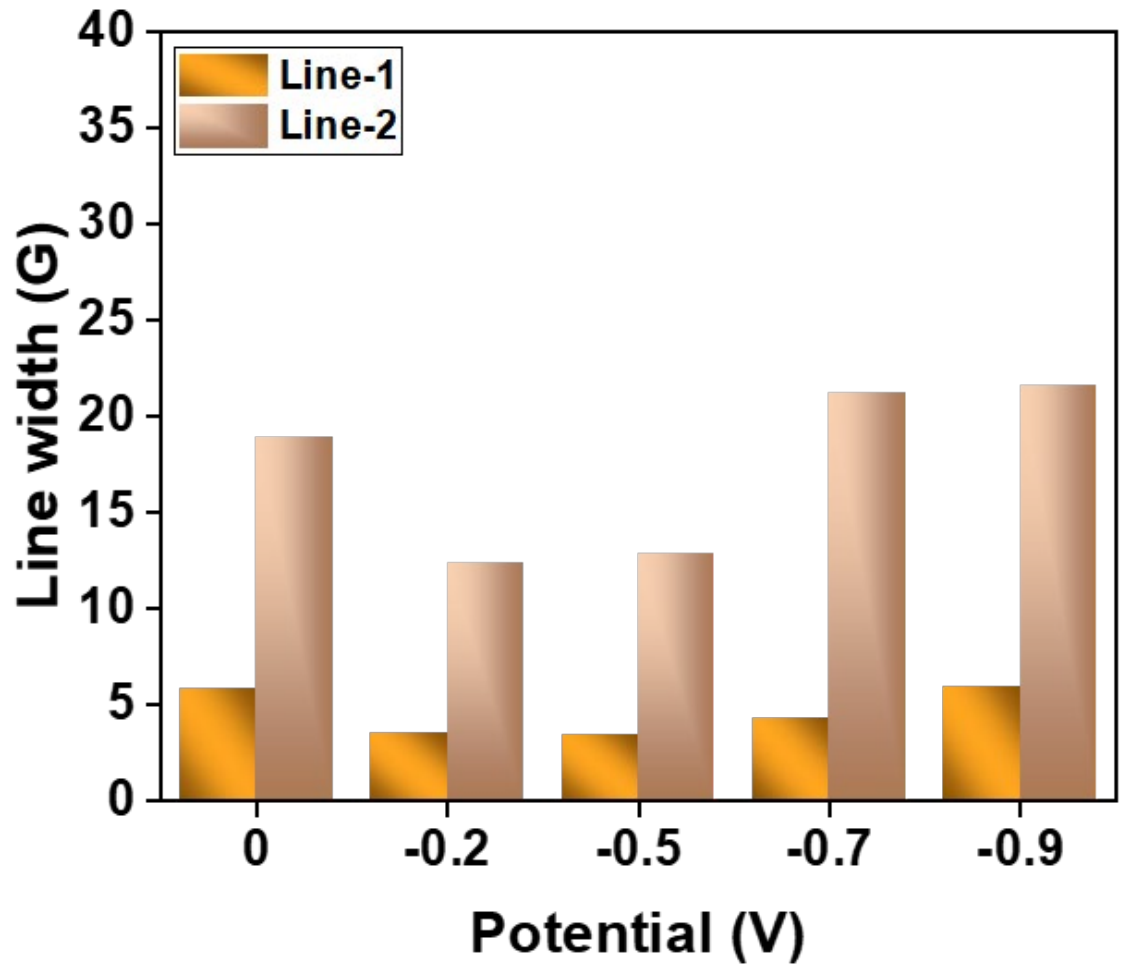


**Supporting Information Figure S13.** The linewidths of Line-1 and 2 from the EPR fittings are plotted as a function of the applied potentials at 20 K. The linewidths of both Line-1 and Line-2 decrease with potential to a minimum at -0.5 V. As potentials further decrease, the linewidths begin to increase and reach their maxima at -0.9 V.

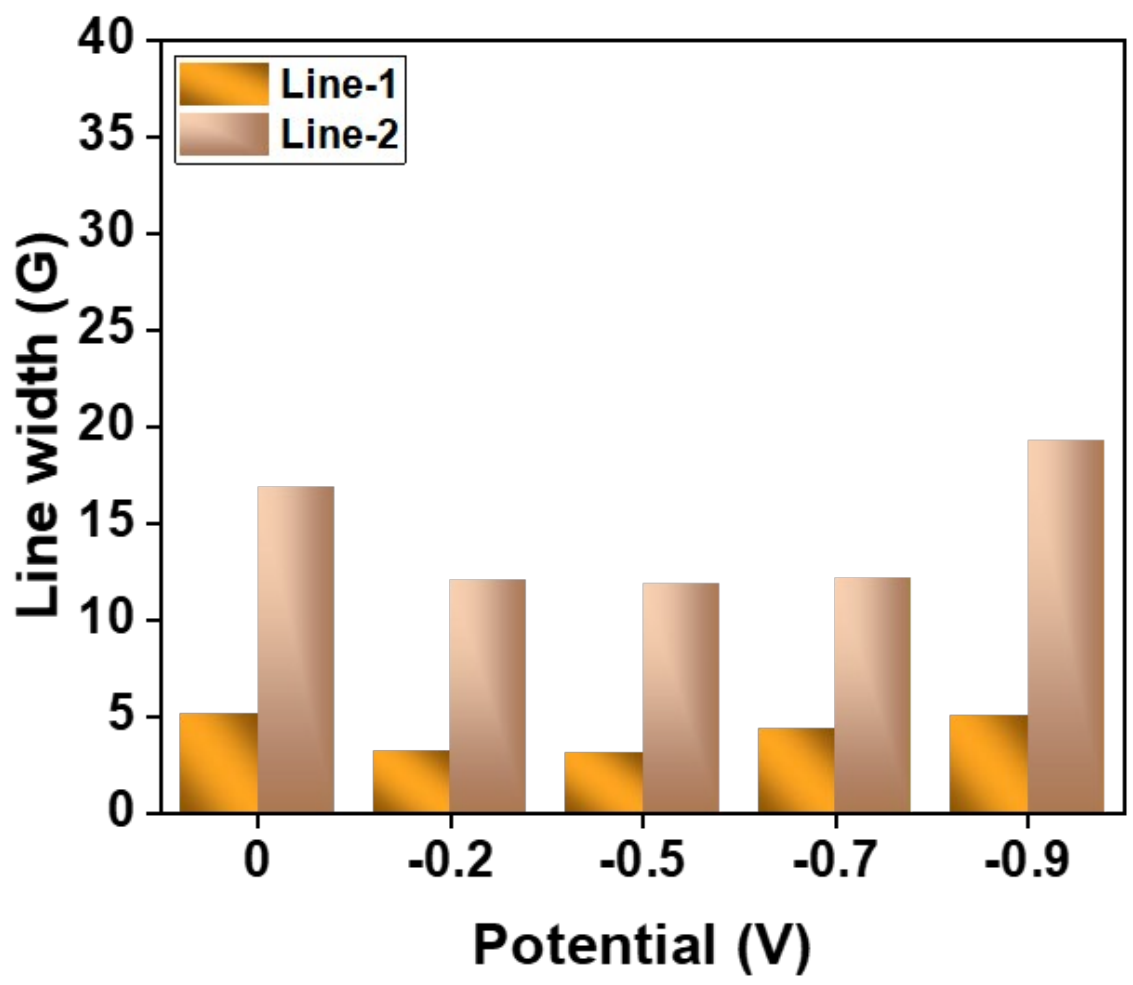


**Supporting Information Figure S14.** The linewidths of Line-1 and 2 from the EPR fittings are plotted as a function of the applied potentials at 30 K. The linewidths of both Line-1 and Line-2 decrease with potential to a minimum at -0.5 V. As the potentials further decrease, the linewidths begin to increase and reach their maxima at -0.9 V.

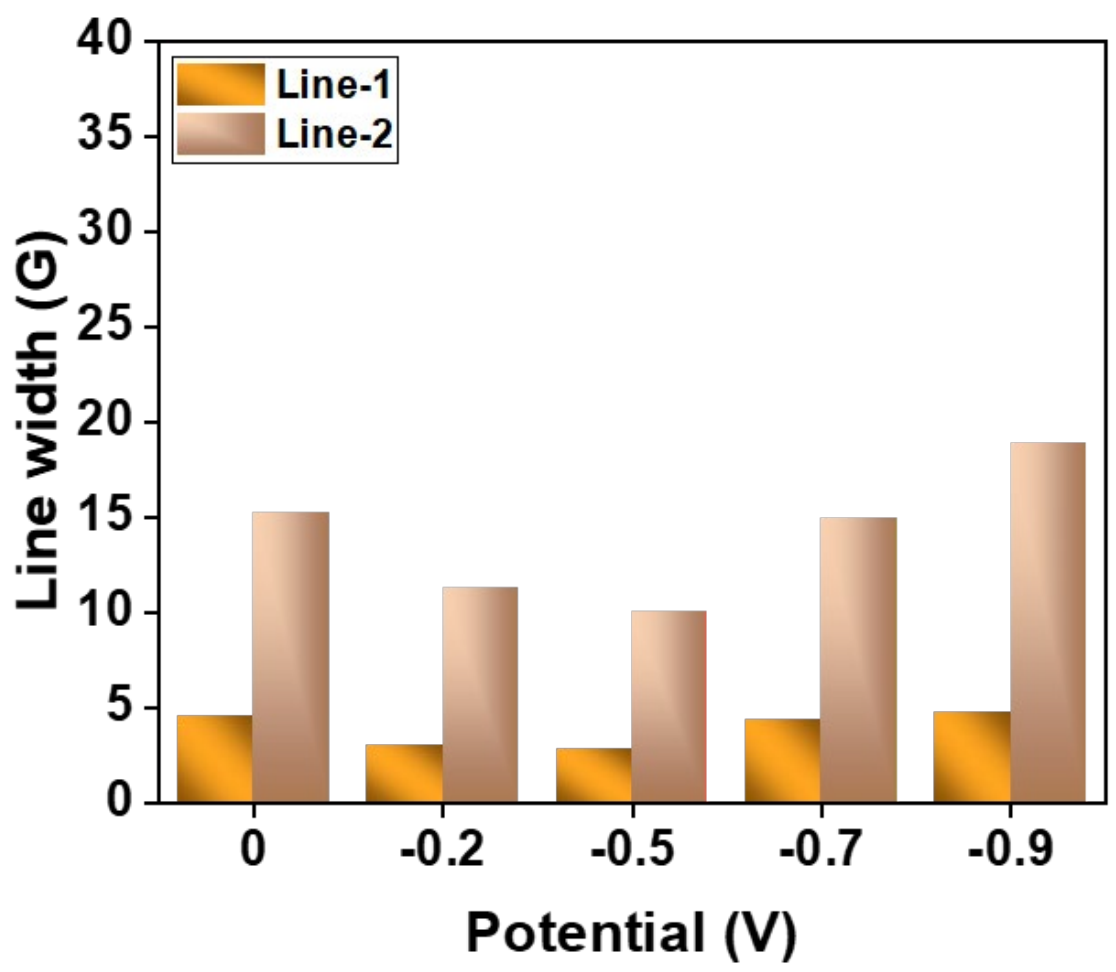


**Supporting Information Figure S15.** The linewidths of Line-1 and 2 from the EPR fittings are plotted as a function of the applied potentials at 40 K. The linewidths of both Line-1 and Line-2 decrease with potential to a minimum at -0.5 V. As the potentials further decrease, the linewidths begin to increase and reach their maxima at -0.9 V.

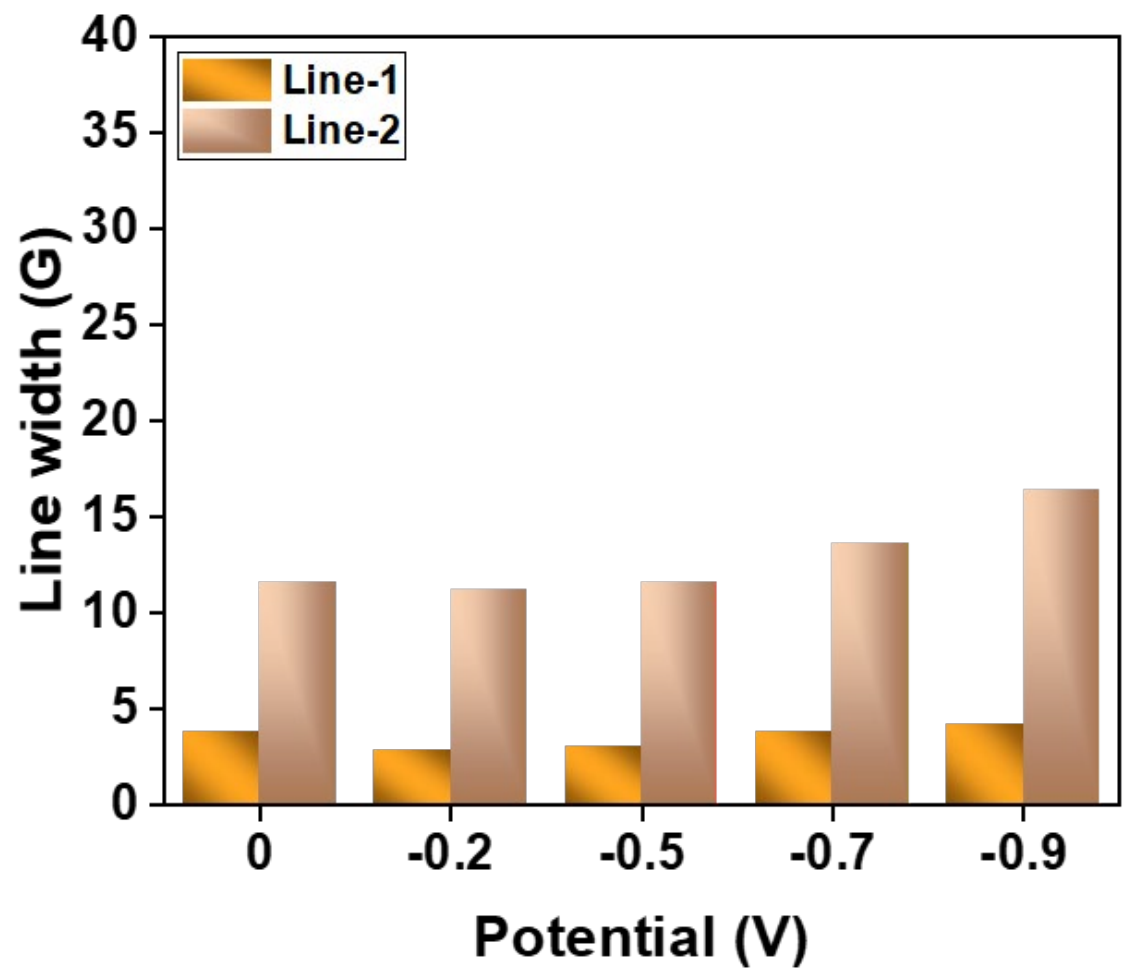


**Supporting Information Figure S16.** The linewidths of Line-1 and 2 from the EPR fittings are plotted as a function of the applied potentials at 50 K. The linewidth of Line-1 decreases slightly with potential to a minimum at -0.5 V. As the potentials further decrease, the linewidth of Line-1 begins to slightly increase and reaches its maximum at -0.9 V. Line-2 appears constant until -0.5 V, and then begins to increase, reaching its maximum at -0.9 V.

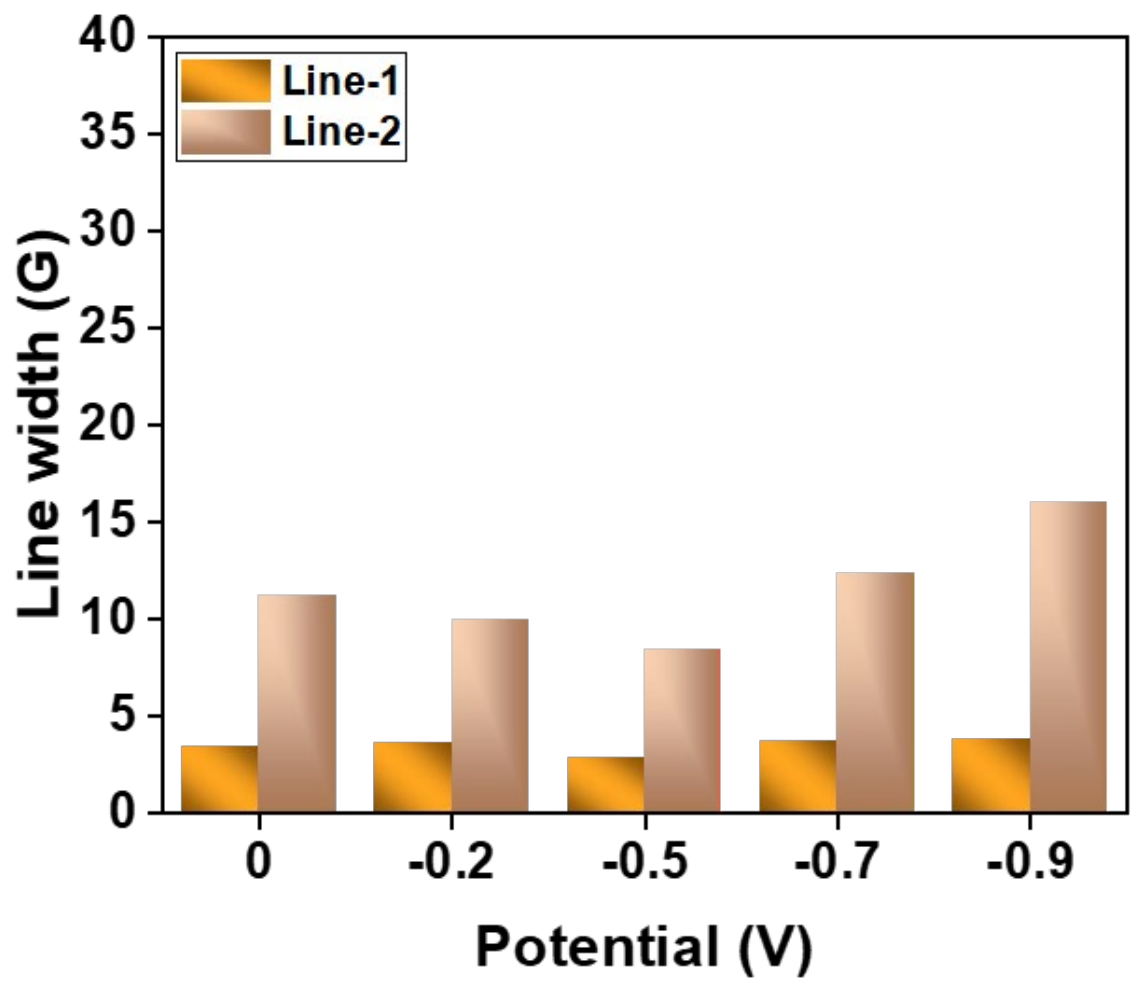


**Supporting Information Figure S17.** The linewidths of Line-1 and 2 from the EPR fittings are plotted as a function of the applied potentials at 60 K. The linewidth of Line-1 remains fairly constant. Line-2 decreases with potential to a minimum at -0.5 V, and as the potential decreases further, its linewidth begins to increase and reaches a maximum at -0.9 V.

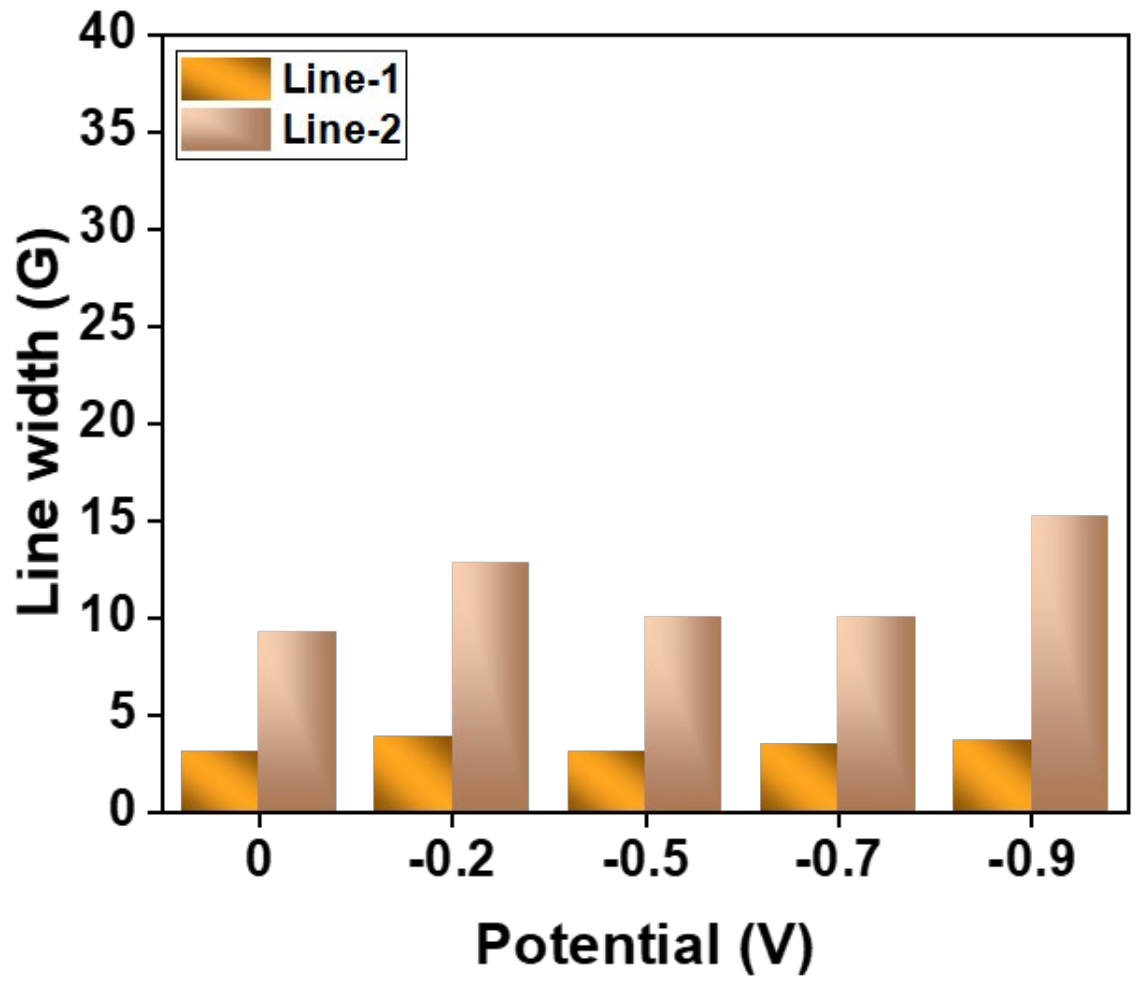


**Supporting Information Figure S18.** The linewidths of Line-1 and 2 from the EPR fittings are plotted as a function of the applied potentials at 70 K. The linewidth of Line-1 remains fairly constant. The linewidth of Line-2 does not exhibit a regular pattern over the potential range.

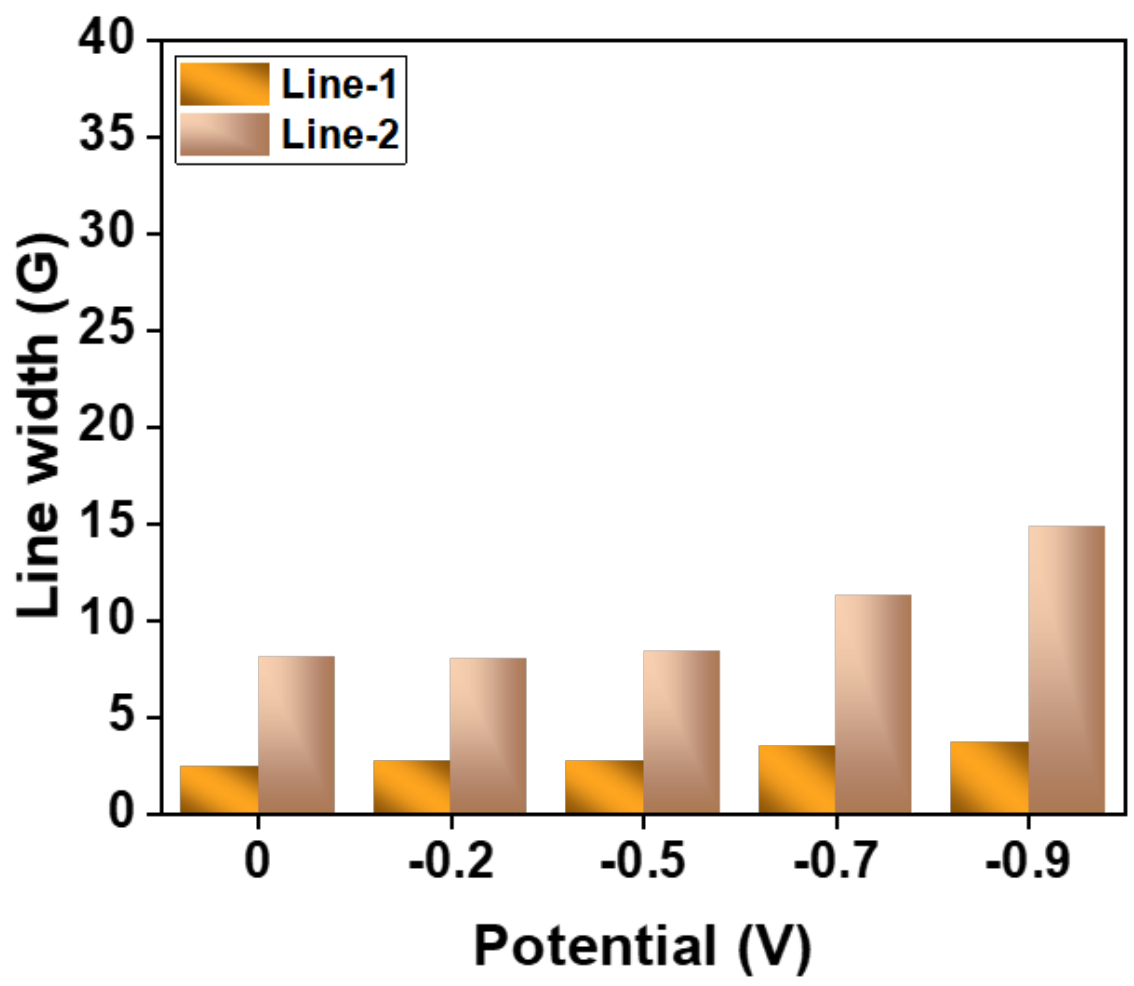


**Supporting Information Figure S19.** The linewidths of Line-1 and 2 from the EPR fittings are plotted as a function of the applied potentials at 80 K. The linewidths of both Lines-1 and 2 remain fairly constant until -0.5 V, and then both exhibit an increase as the potential decreases to -0.9 V.

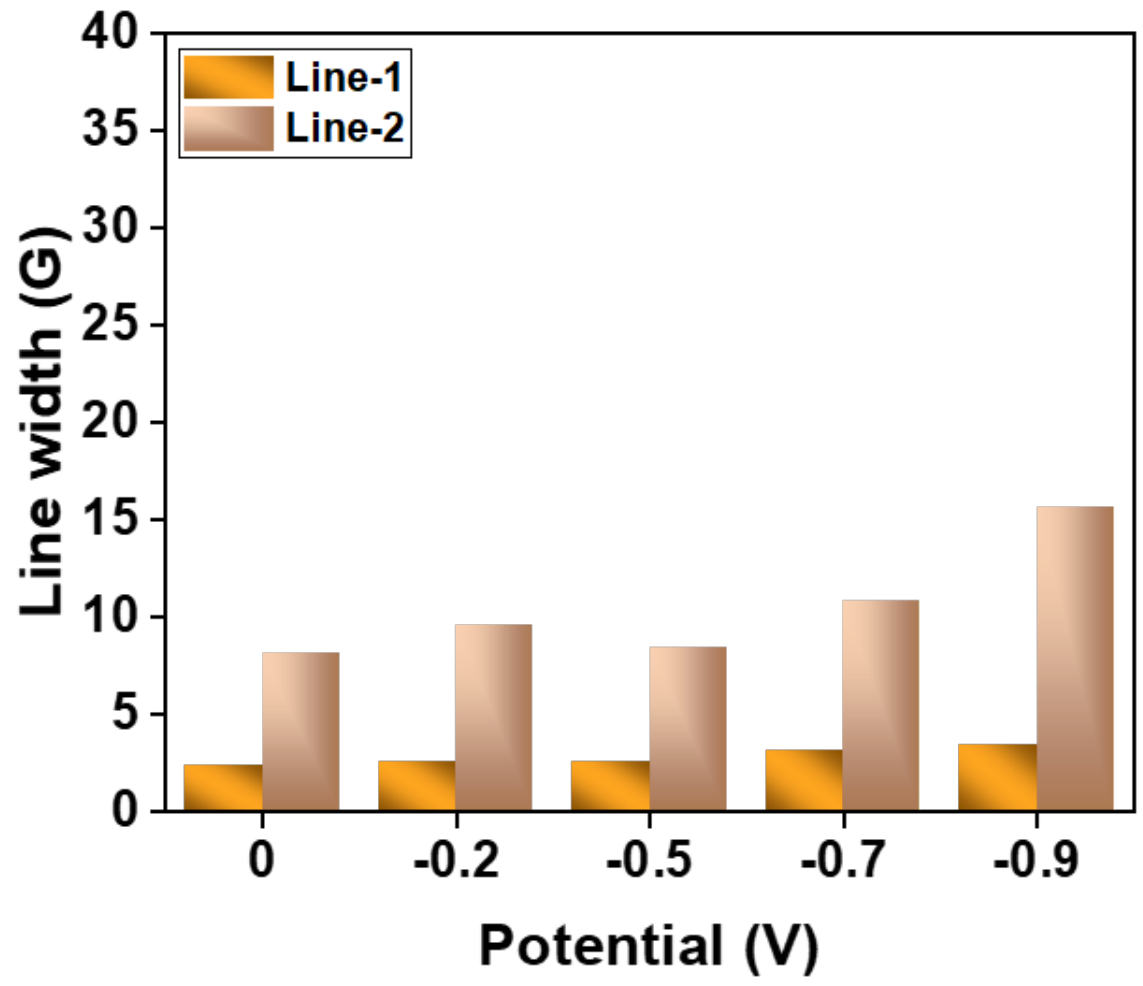


**Supporting Information Figure S20.** The linewidths of Line-1 and 2 from the EPR fittings are plotted as a function of the applied potentials at 90 K. The linewidths of both Line-1 and 2 remain fairly constant until -0.5 V, and then exhibit an increase as the potential decreases to -0.9 V.

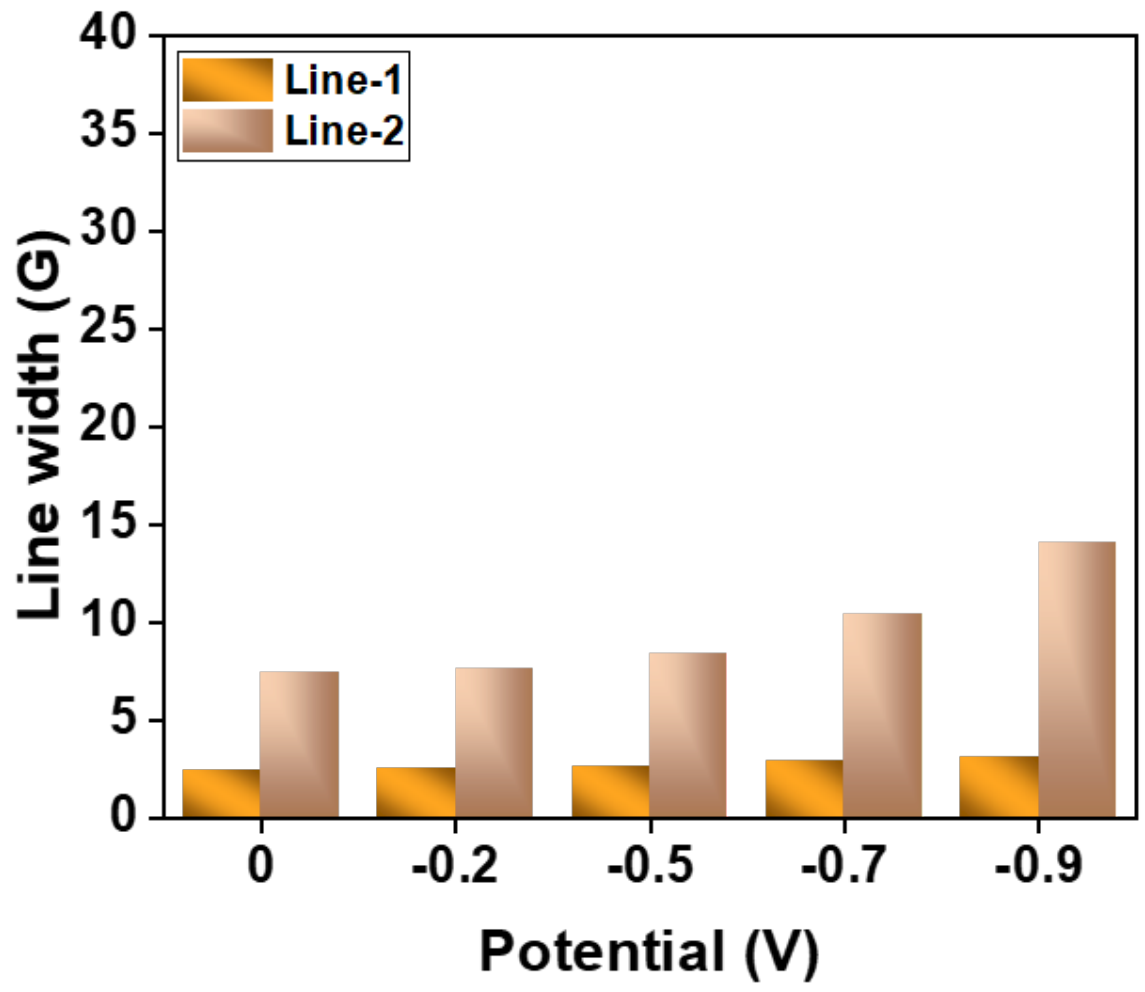


**Supporting Information Figure S21.** The linewidths of Line-1 and 2 from the EPR fittings are plotted as a function of the applied potentials at 100 K. The linewidths of Line-1 remain fairly constant. The linewidth of Line-2 remains constant until -0.5 V, at which point it begins to increase, reaching a maximum at a potential of -0.9 V.
